# Green and Superior Adsorbents Derived from Natural Plant Gums for Removal of Contaminants: A Review

**DOI:** 10.3390/ma16010179

**Published:** 2022-12-25

**Authors:** Hanwen Ge, Ke Ding, Fang Guo, Xianli Wu, Naihua Zhai, Wenbo Wang

**Affiliations:** 1 College of Chemistry and Pharmaceutical Sciences, Qingdao Agricultural University, Qingdao 266109, China; 2 College of Chemistry and Chemical Engineering, Inner Mongolia University, Hohhot 010021, China

**Keywords:** plant gum, adsorbent, contaminants, polymer, wastewater

## Abstract

The ubiquitous presence of contaminants in water poses a major threat to the safety of ecosystems and human health, and so more materials or technologies are urgently needed to eliminate pollutants. Polymer materials have shown significant advantages over most other adsorption materials in the decontamination of wastewater by virtue of their relatively high adsorption capacity and fast adsorption rate. In recent years, “green development” has become the focus of global attention, and the environmental friendliness of materials themselves has been concerned. Therefore, natural polymers-derived materials are favored in the purification of wastewater due to their unique advantages of being renewable, low cost and environmentally friendly. Among them, natural plant gums show great potential in the synthesis of environmentally friendly polymer adsorption materials due to their rich sources, diverse structures and properties, as well as their renewable, non-toxic and biocompatible advantages. Natural plant gums can be easily modified by facile derivatization or a graft polymerization reaction to enhance the inherent properties or introduce new functions, thus obtaining new adsorption materials for the efficient purification of wastewater. This paper summarized the research progress on the fabrication of various gums-based adsorbents and their application in the decontamination of different types of pollutants. The general synthesis mechanism of gums-based adsorbents, and the adsorption mechanism of the adsorbent for different types of pollutants were also discussed. This paper was aimed at providing a reference for the design and development of more cost-effective and environmentally friendly water purification materials.

## 1. Introduction

Water is an indispensable resource, capable of maintaining ecosystems, normal human production, and life. However, a variety of contaminants have been discharged into the environment, which causes serious water pollution and poses a major threat to the safety of the aquatic environment and human health [1,2]. The water pollution problem has aroused widespread concern around the world, and more materials or technologies are needed to effectively eliminate pollutants in water and the hazards they bring [3,4]. Thus far, diversified technologies have been developed for the removal of heavy metals, dyes, antibiotics, phenols, pesticides and other pollutants from water, such as catalytic degradation [5,6], flocculation [7,8], the biological method [9], adsorption [10,11,12,13], filtration [14,15], and demulsification [16]. Catalytic degradation technology, such as photocatalytic degradation [17,18], catalytic oxidation degradation [19,20], and catalytic reduction [21,22,23], etc., can transform organic pollutants into carbon dioxide and water to achieve decontamination. However, this method can do nothing for the pollutants that cannot be degraded, such as heavy metal ions. In addition, secondary pollutants are usually generated during the catalytic degradation process, causing the risk of secondary pollution. The biological method is an industrially used method to eliminate pollutants at present. Activated sludge or microorganisms are used to decompose pollutants [24]. However, the purification rate of the biological method is slow, and it is not suitable for the decontamination of toxic pollutants for microorganisms, such as heavy metals. In contrast, the adsorption method can be widely applied for the removal of various pollutants [25]. The pollutants can be directly “taken out” from water through pore adsorption, electrostatic attraction, ion exchange or other driving forces [26,27,28]. The adsorption process can be applied for the direct purification of pollutants, such as heavy metals, dyes, and antibiotics, without secondary pollution [29,30].

In recent years, the rapid development of material science promotes the development of diversified new adsorption materials. Many adsorbents, such as polymer composites [31,32], ligand immobilized facial composite adsorbents [33], metal organic framework materials [34], superelastic lamellar monoliths [35], mesoporous silicates [36,37,38], polymetallic silicates [39,40], magnetic composites [41,42], biochar [42], and graphene [43], have been developed and applied, which has expanded the application domain of adsorption technology. For example, polymer adsorbents with abundant functional groups can capture more organic molecules (i.e., dyes, antibiotics) or heavy metal ions to achieve the high-capacity adsorption of pollutants [44]. The mesoporous silicate adsorbent with rich pore structure, ion exchange capacity and negative surface charge shows a good removal efficiency for pollutants at low concentration [41,45]. Magnetic composite adsorbents not only show excellent adsorption performance, but also can be easily recycled after use by external magnetic field [46]. With the increasing requirements for the purification of diversified pollutants, adsorbents are also being upgraded from single function to multi-function, and from general adsorption to selective adsorption [47]. With “green development” receiving global attention, the environmental friendliness of adsorbents themselves has also been the focus of attention [48]. While ensuring the good performance of adsorbents, the secondary harm caused by the use of adsorbents should also be minimized [48]. This requires avoiding or reducing the use of toxic or harmful chemicals in the preparation process of adsorption materials. In view of this, using renewable, safe, and low-cost natural materials to develop environmentally friendly adsorption materials has become popular across the world. 

Natural polymers are a kind of biomass resource with renewable, biodegradable, non-toxic merits and abundant reserves in nature. Starch, cellulose, chitosan and natural plant gums are the most well-known natural polymers [49]. They come from green plants or marine organisms on the earth, and have been used as food additives, fine chemical additives, and biodegradable materials [50]. Natural plant gums such as guar gum, xanthan gum, cashew nut gum, and carrageenan gum have also been used as starting materials for synthesis of multiple functional materials [51]. These natural plant gums have good film-forming properties and high strength, and can be easily modified to form degradable polymer materials with improved property and extended application prospects [51,52]. The functional groups (i.e., hydroxyl, carboxyl groups) on the polymer chain of plant gums can generate a grafting polymerization reaction with monomers to form new polymer materials with more functional groups such as carboxyl, sulfonic acid and amino groups [53,54]. New adsorption materials with high mass transfer efficiency and adsorption driving force can also be obtained by the composite of natural plant gum with other organic or inorganic materials [55], and the free radical grafting polymerization reaction [56]. The main or branched macromolecular chains of natural plant gums can be crosslinked together to form a three-dimensional network structure. The massive functional groups in the polymer network can strongly combine with pollutants to achieve a highly efficient removal. The slightly swollen hydrophilic network of plant gums-based polymer materials may reduce mass transfer resistance, and thus improve the adsorption rate. Therefore, the adsorption materials derived from natural plant gums are not only environment-friendly, but also can capture pollutants with a high adsorption capacity and a fast adsorption rate.

This paper comprehensively summarized the research progress on the natural plant gums-derived adsorption materials and their applications in removing pollutants such as heavy metals, dyes, antibiotics, and others. The potential of plant gums-derived materials and environmentally friendly adsorbents were analyzed, and the future development trends were explored. It is expected that this paper will enable readers to fully understand the research status of natural plant gums-based adsorption materials, and provide reference or support for the design and development of more natural polymers-based adsorption materials.

## 2. Natural Plant Gums

Natural plant gums are carbohydrate polymers formed by different monosaccharide units connected with each other through glycosidic bonds [57]. They have diverse physical or chemical properties, such as water solubility, fluidity, thickening, and cohesiveness. The differences of natural plant gums in the composition of the monosaccharide unit, bonding type and polymerization degree cause them to have complex groups and structures with certain electric charges. Some gums, such as xanthan gum, gum arabic, psyllium gum and tragacanth gum, have carboxyl or other functional groups, which belong to anionic polysaccharides. Guar gum mainly possesses the hydroxyl group, which belongs to neutral polysaccharides. These functional groups allow natural plant gums to be modified, so as to introduce more functional groups capable of improving their inherent structure and performance.

## 3. Natural Plant Gums-Derived Adsorbents

A series of adsorbents with excellent adsorption performance for different types of pollutants can be prepared by the modification of natural plant gums. The commonly used modification methods mainly include direct crosslinking [58], composite [59], grafting polymerization [60,61], and grafting-composite [62]. The purpose of modification is to increase the number of active adsorption sites, improve charge distribution, or change pore structure. No matter what method, the improvement of adsorption performance after modification is obvious. Therefore, the preparation methods of the adsorbents derived from different natural plant gums (i.e., guar gum, xanthan gum, pectin gum, carrageenan gum, cashew gum, welan gum, gum arabic), and their adsorption properties for different types of pollutants (such as heavy metal ions, dyes, antibiotics) is summarized in this section.

### 3.1. Guar Gum-Derived Adsorbents

Guar gum is a highly water-soluble natural polysaccharide composed of alternating branched linear chains of mannose and galactose units [63]. The mannose/galactose ratio in guar gum is 2:1, and hydroxyl (-OH) groups exist on the molecular chain [64]. Guar gum can be directly used to remove heavy metal ions in water by taking advantage of its high molecular weight and rich functional groups [65]. The existence of hydroxyl groups on the molecular chain of guar gum makes it an easy gum to be modified with reactive organic molecules or vinyl monomers to form new materials containing more functional groups [66]. The guar gum or its derivative can also compound with other linear polymers or nanomaterials to form composite materials with an improved removal performance of pollutants [67].

#### 3.1.1. Preparation of Guar Gum-Derived Adsorbents

The direct compounding of guar gum or its derivatives with other organic or inorganic components is a commonly used route to prepare guar gum-based adsorption materials. The complementary advantages of different components make it possible to develop new composite materials with an improved comprehensive performance. The other components compounded with guar gum can be directly added with nanoparticles or the particles synthesized in situ in the polymer matrix. For example, the incorporation of the metal organic framework (MOF) materials (i.e., MgMOF74 and CaMOF74) into the crosslinked collagen/guar gum interpenetrating network hydrogel facilitates the increase in the storage modulus of the hydrogel by 324.4 ± 30% (from MgMOF74) and 116.84 ± 10% (for CaMOF74), and also the adsorption performance for dyes and heavy metal ions [66]. The MOF was incorporated into the guar gum matrix by a process described as follows. The MOF particles were dispersed into a collagen solution to form a suspension (1 wt.%), and then the guar gum solution and crosslinker polyurethane was added in turn under continuous stirring. The crosslinking action polyurethane causes a gradual increase in viscosity, until a hydrogel composite with a cross-linking network structure is formed. The composite adsorbents composed of guar gum and other polymers or nanoparticles can be synthesized according to a similar process, that is, a composite structure can be constructed by crosslinking plant gums with other components.

**Figure 1:**
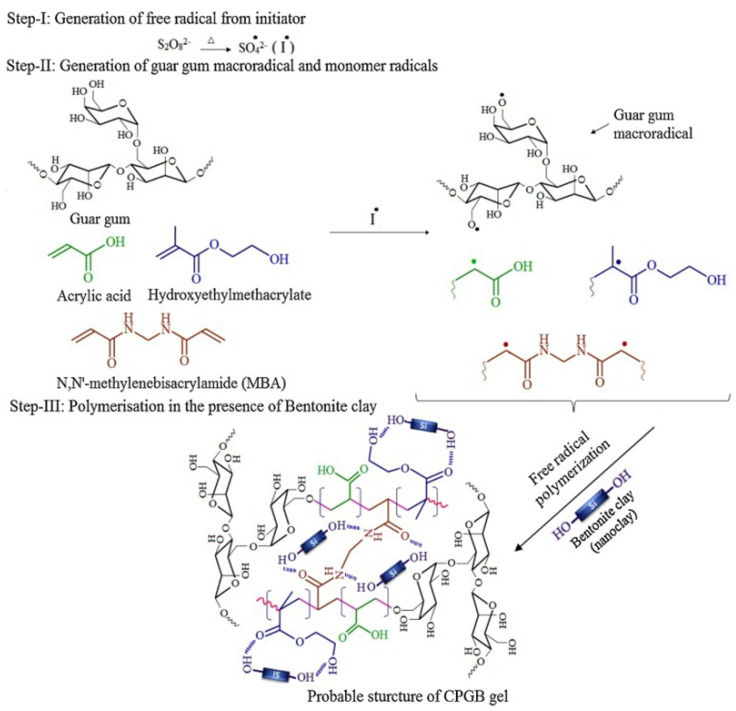
Formation process of guar gum-grafted polymer composite hydrogel [68].

The adsorption capacity or other properties of gums-based adsorbents can be improved by the synergistic effect of different components. In this process, the number of functional groups in guar gum do not increase obviously, so the improvement of adsorption properties is limited. As is known, the electrostatic attraction and chemical complexation of chemical groups play a key role in improving the adsorption of pollutants, such as heavy metal ions. Therefore, introducing more functional groups onto the molecular chain of guar gum is a feasible approach to further improve the adsorption performance. Grafting a polymerization reaction of guar gum with vinyl monomers may introduce large amounts of functional groups into the adsorbent, and thus significantly improve its adsorption capability [67]. During the grafting polymerization reaction process, other components, such as linear polymers or inorganic nanoparticles, can also be incorporated into the polymer matrix to form a polymer composite adsorbent with a better adsorption performance [68]. At present, an aqueous solution graft polymerization reaction is commonly used to synthesize guar gum-grafted polymer materials (Figure 1). The persulfate is usually used as an initiator to ignite a free-radical polymer reaction. The decomposition of initiator generates sulfoxy radicals, which may react with the hydroxyl groups on guar gum to form macromolecular free radicals. Simultaneously, the sulfoxy radicals also attack vinyl monomers to form free radicals with one mono electron. These free radicals in the reaction system may react with each other to complete the chain propagation reaction. The cross-linking agent also participates in the reaction to link the polymer chains to form a network structure [68]. In the presence of inorganic components, such as bentonite, the graft polymerization reaction occurs among guar gum, monomers and other organic or inorganic components. For example, a composite hydrogel adsorbent with excellent removal efficiency for Cr(VI) (removal rate: 97.8% for Cr(VI) in 5 mg/L solution and 91.4% for Cr(VI) in 200 mg/L solution) was synthesized by a grafting polymerization reaction among hydroxyethyl methacrylate, acrylic acid, guar gum and bentonite [68]. 

**Figure 2:**
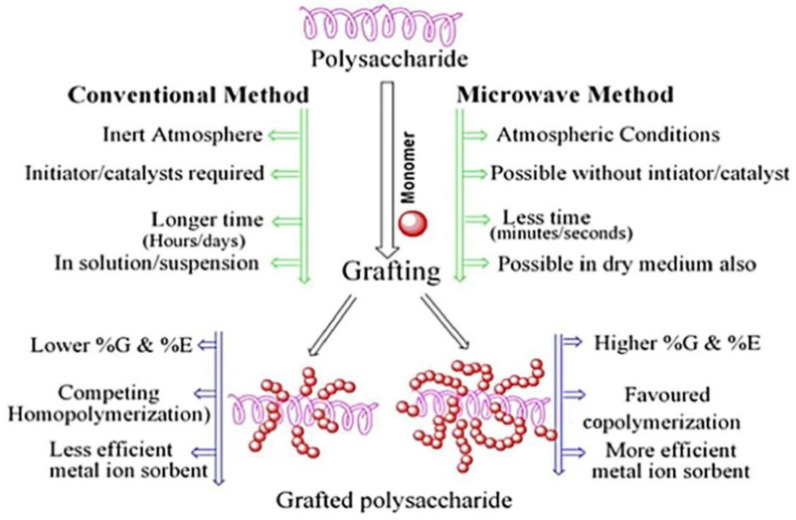
A comparison between conventional graft polymerization and microwave induced graft polymerization routes [70].

Compared with the conventional graft polymerization route, the microwave induced graft polymerization route usually has a faster reaction rate and higher graft efficiency. For example, the monomer ethyl acrylate can be grafted onto guar gum by a microwave irradiation process using potassium persulfate as the initiator [69]. The graft polymer adsorbent shows a satisfactory adsorption capability for pollutants. Batouti et al. [70] compared the advantages of microwave initiated graft polymerization with conventional methods (Figure 2). The monomer acrylamide was grafted onto the macromolecular chain of guar gum by a microwave initiated polymerization reaction process using ammonium persulfate as an initiator. The resultant polymer adsorbent shows an adsorption capacity of 54.054 mg/g for acid red 8. The aqueous solvent can effectively absorb microwave radiation to quickly generate heat energy, so that the initiation reaction can be carried out more efficiently, and so the grafting polymerization reaction rate is higher than the conventional route [71]. In addition, the other properties of the grafted polymer adsorbent, such as mechanical and thermal properties, can also be improved by grafting monomers onto the natural polymers by a microwave-assisted initiation reaction route [72].

#### 3.1.2. Application for Capturing Heavy Metal Ions

Large amounts of wastewaters containing heavy metals lave been discharged from battery, electroplating, production of electronic product, metallurgy and other industrial processes, posing a major threat to the safety of water environments and human health [73,74]. These heavy metals are hardly degraded in the natural environment, and can accumulate in the human body and cause serious diseases such as carcinogenesis and teratogenicity. In recent years, a variety of adsorption materials, such as polymer composites [31,33,75,76], biochar [77,78,79], porous silica [26], MOF [80], cellulose [81], modified magnetite [82], core shell Fe_3_O_4_@polypyrrole composites [83], modified clay minerals [84,85,86], layered double hydroxide [87,88], a magnetite graphene oxide chitosan microsphere [89], magnetic graphene oxide/lignin composites [90], layered metal sulfides [91], PANI@APTS-magnetic attapulgite compounds [92], microcrystalline cell membrane hydroxide [93], and aerogel [94], have been developed to remove heavy metal pollutants in water, and major progress has been made. With the increasing concern regarding the safety of adsorption materials, the synthesis of adsorption materials from natural starting materials has attracted increasing attention [48,95].

The chemical groups on the macromolecular chain of natural guar gum make it easy to be modified to produce adsorption materials with excellent capturing capacity towards heavy metal ions. Chauhan et al. [96] modified guar gum via an acid hydrolysis process in the hydrochloric acid/methanol mixture solution, and then cross-linked it with the crosslinker *N,N*′-methylene-bis-acrylamide to form an adsorption material with an adsorption capacity of 125.893 mg/g for copper ions (adsorbent dosage: 2 g/L; initial concentration: 50 mg/L). The adsorbents with an excellent adsorption capability toward heavy metals can also be prepared by the direct crosslinking of guar gum with other components. Khan et al. [97] prepared a guar gum/nano zinc oxide (GG/nZnO) composite via the in situ loading of zinc oxide in the polymer matrix. The composite can spontaneously adsorb Cr(VI) in an aqueous solution with an adsorption capacity of 55.56 mg/g (initial concentration: 25 mg/L), and can serve as an economic and environmental efficient Cr(VI) adsorbent. The nanocomposite adsorbent composed of guar gum (GG), Fe_3_O_4_@ nickel aluminum layered double hydroxide (Ni/Al LDH)@GG) can efficiently adsorb Cr(VI) with a maximum adsorption capacity of 101 mg/g (pH 6; 30 min) [98]. Guar gum-based composite adsorbents also shows excellent adsorption capability towards radioactive metal ions. The eco-friendly adsorbent derived from phosphorylated guar gum and chitosan can efficiently remove U(VI) from the slightly acidic solution, with the maximum adsorption capacity of 1.28 mmol U/g [99]. The U(VI) adsorbed on the adsorbent can be completely desorbed for recovery. In addition, the adsorbent can be recycled more than five times, and the adsorption performance only loss is only approximately 5–6%. The incorporation of additional magnetite nanoparticles during the preparation process of an adsorbent can produce a composite adsorbent with a satisfactory adsorption capacity of 1.16 mmol U/g and a magnetic separation function.

**Figure 3:**
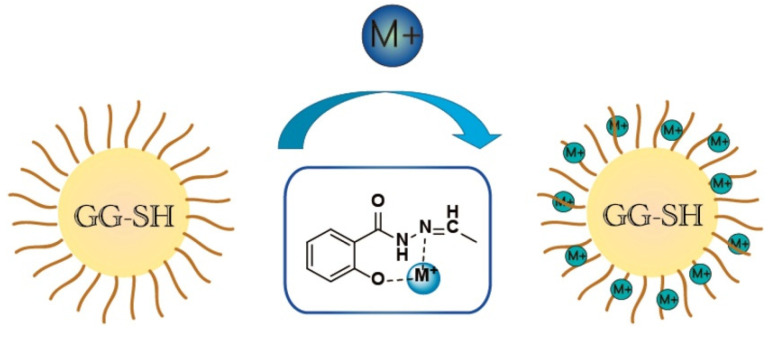
A scheme illustrated the complex process of heavy metal ions by the guar gum-based adsorbent [100].

In principle, the adsorption of heavy metal ions by polymer adsorption materials is mainly driven by electrostatic attraction and the complexation action of functional groups (Figure 3) [100]. Therefore, increasing the number of functional groups on the adsorbent can usually improve the adsorption performance. Grafting polymer chains onto the macromolecular chain of guar gum is an effective way to introduce functional groups and increase the number of adsorption sites of the adsorbent. Ma et al. [100] reported an aldehyde guar gum adsorption material synthesized by grafting salicylic hydrazide onto guar gum. Benefitting from the introduction of functional groups, the grafted polymer material shows very high adsorption capacities of 1272.4, 748.86 and 521.81 mg/g for Ni(II), Cr(III) and Co(II) (initiation concentration: 200 mg/L), respectively. The type and quantity of functional groups on the grafting polymer can be controlled by altering the type or number of monomer, so that the resultant grafting polymer is able to adsorb multiple types of pollutants. The guar gum-graft-poly(acrylic acid-co-acrylamide) adsorbent, prepared by grafting polymerization among acrylic acid, acrylamide and guar gum, shows the maximum static adsorption capacities of 99 mg/g for Cd(II) and 90.3 mg/g for Cu(II) [67]; the adsorption capability did not decrease significantly after the cycle was reused five times. Halim et al. [101] prepared guar gum-graft-poly(acrylamide) using a thiourea dioxide/potassium bromate redox initiation system; then, they cross-linked the grafting copolymer with glutaraldehyde to yield a crosslinked polymer adsorbent capable of efficiently removing Cr(VI) from an aqueous solution. The maximum adsorption capacity of the adsorbent for Cr(VI) reached 588.24 mg/g, which is much higher than that of the unmodified guar gum material [96,97]. Pal et al. [102] synthesized a guar gum-graft-poly(acrylic acid) (GG-g-PAA) adsorbent by a surfactant-mediated free radical polymerization route, which exhibits a high removal rate of 95.32% for Pb(II) in 350 mg/L of the Pb(II) solution (adsorbent dosage: 2.5 g/L).

In addition, to introduce functional groups onto guar gum by a grafting polymerization reaction, the linear polymers or inorganic particles can be introduced to construct interpenetrating networks or composite structures. The guar gum-g-poly(acrylamide-co-acrylamide sodium propionate-co-sodium acrylate) polymer adsorbent with an interpenetrating network structure was prepared by a solution polymerization reaction among guar gum, acrylamide sodium propionate, acrylamide and sodium acrylate [103]. The adsorbent can adsorb Hg(II) with an adsorption capacity of 49.12 mg/g. The polymer composite adsorbent can be prepared by a graft polymerization reaction among guar gum, acrylic acid, hydroxyethyl methacrylate, a cross-linking agent and an inorganic component bentonite, which shows a Cr(VI) adsorption capacity of 182.4 mg/g [69]. The polymer composite adsorbent with a uranium(VI) adsorption capacity of 263.2 mg/g was prepared by a graft polymerization reaction between guar gum and acrylamide in the presence of biochar, which shows a great application prospect for the adsorption and recovery of nuclear pollutants [104]. The guar gum-based adsorption materials also exhibit better capture performance for heavy metal ions under dynamic adsorption conditions. Agata et al. [105] evaluated the adsorption efficiency of a pectin/guar gum biosorbent for zinc ion under dynamic adsorption conditions. At the Zn(II) concentration of 30 mg/L, 2096 mL and 5900 L of Zn(II) solutions can be purified by the small column filled with the adsorbent (2 g fine adsorbent; flow rate of 60 mL/h) and the large column (16 kg large adsorbent; flow rate of 45 L/h), respectively. The adsorbent can be used for more than 20 adsorption-desorption cycles. In addition, the adsorbent can be applied to efficiently remove Zn(II) in the actual wastewater of zinc plating plant at a large scale [105]. In comparison, the preparation process of guar gum-based adsorbents by a direct modification or composite route is simple, while the adsorbent prepared by a grafting polymerization route usually has a better performance.

In addition, to adsorb pollutants in water, guar gum-based adsorbents can also adsorb heavy metals in soil. Subramani et al. [106] used a guar gum-stabilized soil as the liner of the landfill site. It was found that the compressive strength without side walls was increased by 1.75 times, and the hydraulic conductivity after 120 days of curing reduced by 2.72 × 10^5^ times after adding 2% guar gum. In addition, the addition of guar gum significantly increased the retention capacity of soil for lead, copper, cadmium, zinc and other heavy metals. Guar gum was used as a natural coagulant to treat the landfill leachate to prevent secondary environmental pollution [107]. When the dosage of guar gum is 44.39 mg/L (pH 8.56; stirring speed: 79.27), the removal rate of chemical oxygen demand is 22.57%, and the various ions in leachate can be adsorbed by guar gum. The hydrogen bond interaction between guar gum and pollutant particles participates in the coagulation and flocculation process, which proves the application potential of guar gum in landfill leachate treatment.

#### 3.1.3. Application for Capturing Dyes

**Figure 4:**
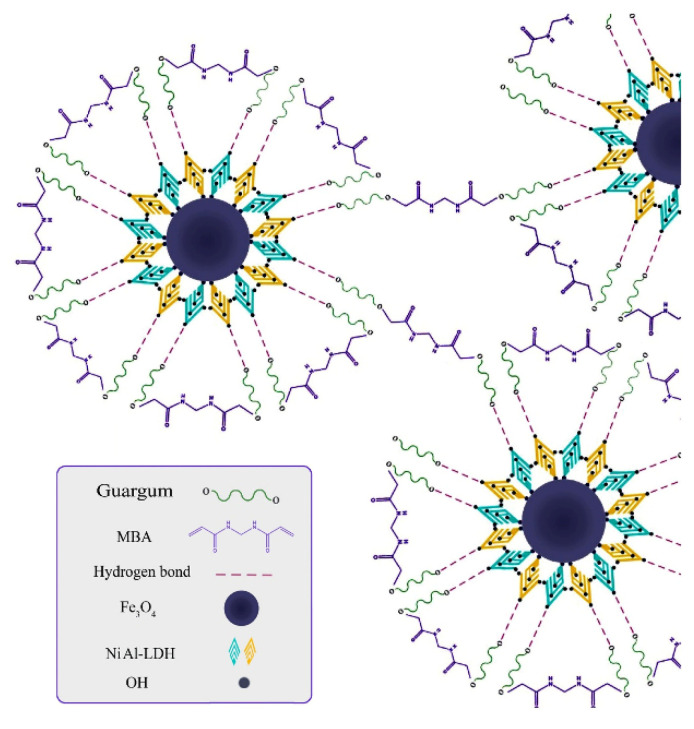
A scheme illustrated the synthesis process of cross-linked GG-NiAlLDH-Fe_3_O_4_ biological nanocomposite [116].

More materials or technologies are urgently demanded to eliminate the pollution caused by dye pollutants [108,109,110,111,112,113]. In recent years, eco-friendly water purification materials prepared from renewable natural polymers have been focused upon [48]. The adsorption materials with excellent dye adsorption properties can be obtained by a composite or the graft polymerization reaction process of guar gum. The biodegradable nanocomposite adsorbent with excellent adsorption capacity of cationic dyes (781.25 mg/g for malachite green and 281.69 mg/g for safranin) (initial concentration: 400 mg/L for malachite green and 100 mg/L for safranin; adsorbent dosage: 1 g/L for malachite green and 0.83 g/L for safranin) can be prepared by the composite of anionic modified guar gum and SiO_2_ nanoparticles [114]. The guar gum/nickel tungstate nanocomposites were prepared by a sol-gel and in situ crosslinking route, which shows the excellent adsorption capacity of 220.21 mg/g for phthalocyanine B (PB) and 170.42 mg/g for crystal violet (CV) (initial concentration: 80 mg/L; adsorbent dosage: 1.5 g/L for PB and 2 g/L for CV) [115]. After use, the adsorbent can be regenerated by a desorption process with a desorption efficiency of 57.60%~91%. The magnetic layered double hydroxide (NiAlLDH-Fe_3_O_4_) was incorporated into guar gum to form the biological nanocomposite GG-NiAlLDH-Fe_3_O_4_. The NiAlLDH-Fe_3_O_4_ particle was embedded into the crosslinked guar gum network by a hydrogel-bonding interaction to form a composite adsorbent with an adsorption capacity of 64.5 mg/g (initial concentration: 200 mg/L) and a removal rate of 84% (initial concentration: 50 mg/L; adsorbent dosage: 1.8 g/L) (Figure 4) [116]. The binary organic composites (carboxymethyl cellulose/guar gum) and ternary inorganic organic nanocomposites (copper oxide/carboxymethyl cellulose/guar gum) were prepared by a composite route of multiple components [117]. The adsorption capacity of the CMC/GG/CuO−3 adsorbent towards malachite green dye is 18.5 mg/g. Similarly, a biopolymer nanocomposite with an adsorption capacity of 17.6 mg/g and removal rate of 88.2% for malachite green was prepared by compounding carboxymethyl cellulose, guar gum, and graphene oxide [118]. The composite adsorbent prepared by incorporating porous materials, such as activated carbon into the guar gum substrate, also show a good adsorption capability towards anionic dyes. The guar gum/activated carbon composite adsorbent can spontaneously adsorb 64.47 mg/g of Congo red dye (initial concentration: 50 mg/L; adsorbent dosage: 0.5 g/L) [119], and can be reused for more than five continuous cycles. 

In order to further improve the adsorption performance of guar gum-based adsorption materials and their applicability to different types of dyes, the graft polymerization and composite routes are often used to introduce more functional groups or components to improve the capturing ability of adsorbents for pollutants. Nanoparticles are the commonly used functional components capable of improving the performance of adsorption materials [120]. Singh et al. [121] prepared a guar gum-graft-poly(acrylic acid)/silver nanoparticle composite with an excellent adsorption capacity for methylene blue (833.33 mg/g) via the in situ incorporation of a silver nanoparticle (size: 100 ± 1 nm) in the polymer matrix. The hydrogen bond interaction and electrostatic attraction between the carboxyl group of adsorbent and the N, S group of methylene blue are the main driving forces for the adsorption process.

#### 3.1.4. Application for Capturing Bactericide

The materials capable of removing bactericides from water have received increasing attention in recent years [122,123,124], but few studies have focused on the bactericide adsorption materials derived from natural polymers. Sharma et al. [125] prepared a guar gum crosslinked soybean lecithin hydrogel film for the effective removal of fungicides and methyl thiophenate from an aqueous solution. The highly active functional groups on the surface of the film make it capable of adsorbing spontaneously bactericide with a better adsorption efficiency. The adsorption capacity of the adsorbent for sodium methylthiosulfate reaches 59.205 mg/g (initial concentration: 25 mg/L). With the extensive use of bactericides and antibiotics, the water pollution caused by these pollutants will receive increasing attention. Natural polymers-based adsorption materials will play an important role in the decontamination of these pollutants.

#### 3.1.5. Application for Capturing Pesticides

The water pollution caused by the excessive use of pesticides has received more and more attention [126,127,128,129,130,131,132,133,134]. However, few adsorption materials derived from natural plant gum were developed for pesticide removal. Nikzad et al. [135] prepared a magnetic guar gum adsorbent with a particle size distribution of 56~92 nm and a magnetic guar gum/montmorillonite nanocomposite adsorbent with a particle size distribution of 70~128 nm by a coprecipitation route. The adsorption capacity of magnetic guar gum and magnetic guar gum/montmorillonite composite for pesticide diazinon are 47.17 mg/g and 80.00 mg/g, respectively. Pesticides are toxic pollutants that can cause severe harm to human health even at a low concentration. Therefore, the development of new natural plant gum-based adsorption materials capable of effectively eliminating such pollutants has broad application prospects.

#### 3.1.6. Application for Oily Wastewater Purification

The waste pollution caused by petroleum and organic solvents has become a global environmental problem. Superhydrophobic materials have been developed to solve this problem. However, these materials are easily polluted by high viscosity oil, leading to a decline in the separation efficiency. Therefore, oil–water separation materials, with high hydrophilicity and hydrophobicity in air, must be developed via a simple and economical route. Wang et al. [136] prepared a guar hydroxypropyl trichloro perfluoroanonanoate/kaolin coated nylon fabric via an impregnation coating method. The introduction of cationic guar gum and kaolin gives the coated fabric superhydrophilicity, good air oil repellency and low viscosity; therefore, it can resist water and oil, and can be used to separate a water/oil mixture in acid, alkaline, salt solution and hot water with an oil-water separation efficiency higher than 96.7%. The grafted guar gum can also be used as a multi-functional material for oil-water separation and adsorption of metal ions. Wen et al. [137] prepared a multifunctional poly(methacryloyl hydrazone) modified guar gum adsorbent via the reaction of hydrazine hydrate-modified poly(acrylic acid-*co*-methyl methacrylate) with dialdehyde guar gum. This material can not only efficiently separate oil from water, but also efficiently adsorb dyes and heavy metal ions with the adsorption capacities of 1418.36 mg/g for methylene blue, 1375.58 mg/g for malachite green, and 196 mg/g for Cu(II) (adsorbent dosage: 1 g/L; initial concentration: 1000 mg/L for dyes, and 800 mg/L for Cu(II)), which greatly expands the application field of guar gum-based adsorption materials.

### 3.2. Xanthan Gum-Derived Adsorbents

Xanthan gum is a renewable extracellular high molecular weight polysaccharide produced from a natural bacterium. It consists of two glucose units, two mannose units and a repeating five pond unit of a glucuronic acid unit. Its chemical structure is similar to cellulose, and its main structure is a linear (1–4) connected glucose, similar to the main chain of cellulose. It has many functional groups (i.e., carboxyl and hydroxyl groups) that can be modified to enhance its physical and chemical properties [138]. It shows significant potential in many fields such as biomedicine, wastewater treatment, the food industry, and agriculture [139]. The xanthan gum is negatively charged, which can attract positively charged molecules or ions by electrostatic attraction and the chemical complexing interaction. The xanthan gum-based adsorption materials can be prepared via many routes, such as direct crosslinking [140], graft polymerization [141], and composite [142]. The hydroxyl group on xanthan gum can react with reactive organic molecules, such as maleic anhydride and chloroacetic acid, to introduce functional groups, and can also react with vinyl monomers to introduce side chains with large amounts of functional groups. The monomers can be grafted onto xanthan gum by a free radical polymerization reaction (Figure 2). In recent years, the ultrasonication, microwave and ray assisted graft polymerization reaction has also been employed to improve the polymerization reaction efficiency [142]. In addition, various new initiation systems have been developed to improve the polymerization reaction efficiency of xanthan gum and enhance the performance of the resultant materials [143].

Ultrasonic action may promote the polymerization reaction of xanthan gum with vinyl monomers and improve the dispersion of inorganic components in the polymer substrate; this improves the uniformity and comprehensive performance of the resultant adsorption materials. Motshabi et al. [142] prepared a xanthan gum-graft-poly(acrylic acid-*co*-itaconic acid)/zinc oxide nanocomposite hydrogel with the assistance of ultrasonication action. The hydrogel shows an adsorption capacity of 212.8 mg/g for methylene blue (initial concentration: 400 mg/L; adsorbent dosage: 0.5 g/L), and the addition of ZnO nanoparticles enhances the adsorption capacity of the hydrogel for methylene blue by 14.6%. Based on the conventional persulfate initiation system, researchers have also made an effort to develop new initiation systems to improve the graft polymerization efficiency. Kaur et al. [143] prepared a xanthan gum-graft-poly(acrylamide-co-N-hydroxymethylacrylamide-co-N,N-dimethylacrylamide-co-acrylic acid) using thionyl chloride as the initiator and promoter. The thionyl chloride can form ion pairs on the xanthan gum to fully activate it. Then, the monomers attack the positively charged center to conduct the graft polymerization reaction, and the xanthan-grafted copolymer can be formed. The adsorption material prepared by this reaction route shows a better adsorption performance for pollutants, with adsorption capacities of 119.27 mg/g and 122.67 mg/g, and a removal rate of 99% and 97% for cationic malachite green and anionic monochrome black T, respectively. This result proves that the thionyl chloride induced cationic polymerization reaction is a promising way to prepare natural plant gums-grafted polymer adsorbents.

#### 3.2.1. Application for Capturing Heavy Metals

It is urgent to develop more materials to resolve the water pollution problem caused by heavy metal ions [144,145]. Xanthan gum shows relatively stronger chelating ability to heavy metal ions due to the presence of carboxyl groups. The adsorption capacity of xanthan gum towards heavy metal ions can be improved by directly modifying xanthan gum with reactive organic molecules or vinyl monomers. Qiu et al. [146] prepared an adsorbent with an adsorption capacity of 46.95 mg/g for Cu(II) (adsorbent dosage: 2 g/L; initial concentration: 885 mg/L) and reusability by the modification of xanthan gum with thionyl chloride and ethylenediamine. Xanthan gum or its derivatives can integrate with other components to form adsorption materials with a strong adsorption capacity for heavy metal ions. Peng et al. [147] prepared a magnetic Fe_3_O_4_@silicon dioxide/xanthan gum composite for the removal and recovery of Pb(II) from water. The carboxyl groups on xanthan gum provide adsorption sites for selectively capturing Pb(II) ions (adsorption capacity: 21.32 mg/g) (adsorbent dosage: 0.5 g/L; initial concentration: 2 mg/L). The Pb(II) ions adsorbed by the adsorbent can be desorbed with 0.05 mol/L hydrochloric acid solution and recovered, suggested a great application prospect of the adsorbent for the purification of battery industrial wastewater and recovery of Pb(II). Xanthan gum is a green candidate for the preparation of environmentally friendly adsorption materials. Mirza et al. [148] synthesized a biodegradable xanthan gum/montmorillonite nanocomposite adsorbent with an excellent adsorption capacity of 130 mg/g for Pb(II) (initial concentration: 100 mg/L). After adsorption with 0.5 g/L of the nanocomposite adsorbent, the concentration of Pb(II) in electroplating and battery manufacturing wastewaters decreased from 2.6 to 0.5 mg/L (removal rate: 80.77%), and from 8.5 to 2.5 mg/L (removal rate: 70.58%), respectively. Except for montmorillonite, modified mica can also combine with xanthan gum to yield adsorption materials with better adsorption performances. Ahmad et al. [149] prepared an eco-friendly nanocomposite (xanthan gum/n-acetylcysteine mica) with an adsorption capacity of 530.54, 177.2 and 51.48 mg/g for Pb(II), Cu(II) and Ni(II) (initial concentration: 100 mg/L), respectively. The metal ions adsorbed on the adsorbent can be desorbed with hydrochloric acid solution, and the metals can be recovered for reuse. The preparation process of a composite adsorbent by the combination of xanthan gum with other components is simple, and no or few harmful chemicals were used in the preparation process, so this route shows a great prospect in the development of cost-efficient adsorbents for practical application.

**Figure 5:**
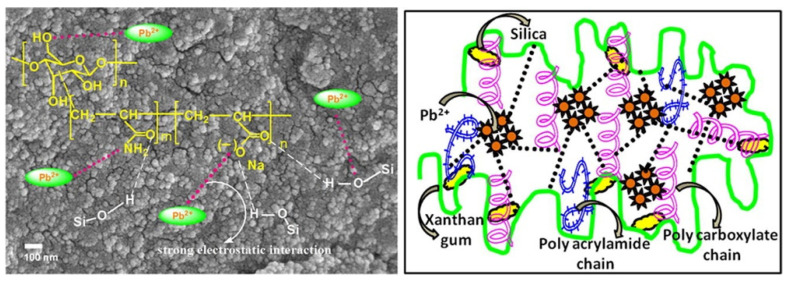
Morphology and structure of xanthan gum-*g*-poly(acrylamide)/nano silica composite adsorbent and its adsorption mechanism for Pb(II) ion [152].

It is often necessary to introduce more functional groups onto the macromolecular chains of the xanthan gum chain for efficiently removing different types of metal ions, which can be achieved by a graft polymerization reaction. Sharma et al. [141] synthesized a xanthan gum-cl-poly(acrylamide-co-alginate) hydrogel containing carboxyl, amide and hydroxyl groups by a microwave-assisted graft copolymerization reaction among xanthan gum, acrylamide, and alginic acid. The introduction of functional groups promotes the adsorption and removal of harmful Cd(II) ions from water by the adsorbent, with an adsorption capacity of 125 mg/g (initial Cd(II) concentration: 60 mg/L; adsorbent dosage: 0.5 g/L). Pandey et al. [150] prepared a xanthan gum-grafted polymer adsorbent by a microwave-promoted emulsion copolymerization reaction between ethyl acrylate and xanthan gum. The introduction of functional groups on the xanthan gum promotes the rapid and efficient adsorption of Pb(II) (adsorption capacity: 142.86 mg/g; rate constant: 3.013 × 10^−4^ g/(mg·min)) (Pb(II) concentration: 100 mg/L). During the graft polymerization reaction process, other components can be incorporated into the polymer network to form a composite adsorbent with a performance better than a neat polymer adsorbent. Ghorai et al. [151] prepared a xanthan gum-graft-polyacrylamide/silicon dioxide composite adsorbent with a high adsorption capacity of 537.634 mg/g towards Pb(II) (adsorbent dose: 1 g/L; initial concentration: 1000 mg/L), which shows great potential for the purification of battery industrial wastewater and the recovery of valuable metal ions such as Pb(II). The research group also prepared a partially hydrolyzed xanthan gum-*g*-poly(acrylamide)/nano silica composite adsorbent for the efficient and rapid removal of toxic Pb(II) ions in water [152]. Thanks to the strong electrostatic interaction and the chelation action of the adsorbent with Pb(II) (Figure 5), the composite adsorbent shows a high removal rate of 99.54% within 25 min (initial concentration: 600 mg/L; adsorbent dosage: 1 g/L) and an adsorption capacity of 1012.15 mg/g for Pb(II) (initial concentration: 1200 mg/L; adsorbent dosage: 1 g/L). The excellent performance of the adsorbent gives it potential to be used for the decontamination of Pb(II) in battery industrial wastewater.

#### 3.2.2. Application for Capturing Dyes

Xanthan gum-based adsorbents are environmentally friendly and have great potential to be used for the effective removal of toxic dyes from industrial wastewater. Xanthan gum-based adsorbents may capture dyes through electrostatic interaction, hydrogen bond interaction and π-π interaction [143], so their adsorption capability can be improved by optimizing the type or number of chemical groups on xanthan gum. For example, the direct modification of xanthan gum with maleic anhydride may increase the number of carboxyl groups on the macromolecular chain of xanthan gum, and thus improve its adsorption performance for methylene blue (adsorption capacity: 435 mg/g) (initial concentration: 800 mg/L; adsorption dosage: 1.2 g/L) [153]. The reaction among xanthan gum, maleic anhydride and hydroxyapatite. in a certain proportion, formed a composite adsorbent capable of adsorbing methylene blue in water, with a high adsorption capacity of 769 mg/g (initial dye concentration: 1000 mg/L; adsorbent dosage: 0.4 g/L) [154]. The introduction of carboxyl groups intensified the electrostatic attraction and hydrogen bonding interaction between the adsorbent and dyes, resulting in a significant increase in the adsorption capacity. The xanthan-based adsorbents can be prepared as monolith materials to make them easily recyclable after use, which is advantageous over the conventional powder adsorbents. Liu et al. [155] prepared a xanthan gum/graphene oxide three-dimensional aerogel via an ice crystal template method at −40 °C. The aerogel not only shows a high adsorption capacity of 244.36 mg/g and 290.57 mg/g, and a removal rate of 95% and 97% for rhodamine B and methylene blue (initial concentration: 100 mg/L; adsorbent dosage: 0.1 g/L), respectively, but also can be easily separated from the solution. The aerogel adsorbent can be reused, and its adsorption efficiency for rhodamine B and methylene blue can continue to reach 88% and 90% after being reused in nine cycles, respectively.

The direct composite of xanthan gum with other components is simple, but the resultant adsorbents cannot meet the requirement for the high-efficiency purification of various pollutants. Therefore, more functional groups with a stronger chelating capacity must be introduced to pollutants by grafting a different type of monomer onto the macromolecular chains of xanthan gum. Chaudhary et al. [156] synthesized a xanthan gum-psyllium-cl-poly(acrylic acid-*co*-itaconic acid) polymer adsorbent with an increased number of functional groups. The introduction of carboxyl groups improved the electrostatic attraction, dipole–dipole, and hydrogen bonding interaction between adsorbent and adsorbate, and thus enhanced the removal rates of dyes auramine-O (initial concentration: 15 mg/L; adsorbent dosage: 15 g/L) and eriochrome black-T (initial concentration: 30 mg/L; adsorbent dosage: 15 g/L) by the adsorbent (95.63% and 90.53%, respectively). Njuguna and Schönherr [157] synthesized a xanthan gum-graft-poly(acrylic acid) hydrogel with a nice adsorption capability towards the cationic dye gentian violet. The slight swelling property of the hydrogel may reduce the mass-transfer resistance and increase the adsorption driving force, and the introduced functional carboxyl groups may increase the electrostatic attraction between the adsorbent and cationic dyes, resulting in a high adsorption capacity of 502 mg/g (initial concentration: 600 mg/L; adsorbent dosage: 1.2 g/L). In addition, the anionic character of the hydrogel adsorbent enables it to selectively adsorb gentian violet in the binary mixture of gentian violet/methyl orange. In recent years, with the increasing requirements for wastewater treatment, it is expected to kill or remove microorganisms in water while removing pollutants. As mentioned above, the other functional components can be introduced during the graft polymerization reaction process, which make it possible to develop multi-functional adsorption materials. Elella et al. [61] prepared a xanthan gum-graft-poly(N-vinyl imidazole) adsorbent via a grafting polymerization and crosslinking reaction. The adsorbent is multi-functional, which can not only efficiently adsorb crystal violet (removal rate: 99.7%; adsorption capacity: 625 mg/g) (initial concentration: 500 mg/L; adsorbent dosage: 0.8 g/L), but can also kill or remove bacteria in wastewater. The incorporation of other filling components into a xanthan gum-grafted polymer can further improve its comprehensive properties. Motshabi et al. [142] prepared a xanthan gum-graft-poly (acrylic acid-co-itaconic acid)/zinc oxide hydrogel adsorbent via a ultrasonication-assisted polymerization reaction process. The incorporation of ZnO enhanced the adsorption capacity of the adsorbent for methylene blue by 14.6%. Taktak et al. [158] prepared a xanthan gum-cl-2-(N-morpholinoethyl methacrylate)/titanium oxide composite with a special interpenetrating network and nanocomposite structure. With benefits from the polar oxygen-containing functional groups (i.e., -OH, C=O, C-O-C, and -COOH), the adsorbent can effectively adsorb methylene blue and crystal violet dyes with the adsorption capacities of 63.34 mg/g and 87.25 mg/g, respectively (initial concentration: 300 mg/L; adsorbent dose: 0.4 g/L). Hosseini et al. [159] prepared a xanthan gum/poly(acrylic acid)/hydroxyapatite composite aerogel with a semi-interpenetrating network structure, which may efficiently adsorb methylene blue (adsorption capacity: 130 mg/g) (initial concentration: 200 mg/L; adsorbent dose: 1 g/L) via a strong hydrogen bonding and electrostatic interaction. The removal rate of methylene blue remains above 86% after ten rounds of recycling. Wang et al. [160] synthesized a xanthan gum-g-poly(acrylic acid-co-acrylamide)/biochar composite hydrogel via a free radical graft polymerization reaction in an aqueous medium. The introduction of biochar improved the roughness and pore structure of the hydrogel, and thus enhanced the mass-transfer driving force to achieve the efficient and fast adsorption of dye (removal rate: 96.64%) (adsorbent dose: 1 g/L; initial concentration: 14 mg/L).

#### 3.2.3. Application for Capturing Other Pollutants

**Figure 6:**
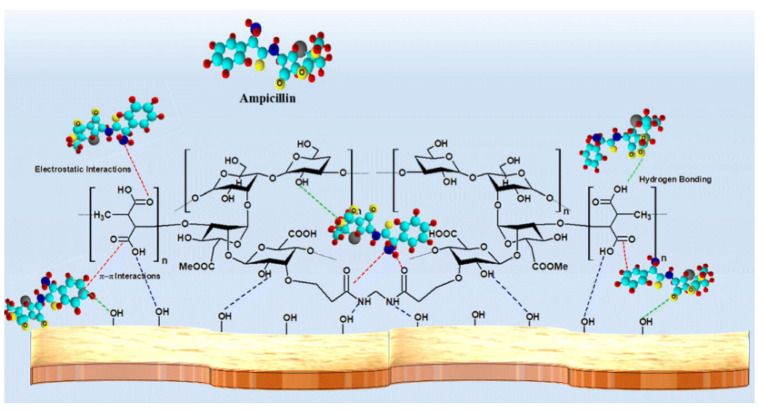
Adsorption of ampicillin onto the xanthan-based composite adsorbent [167].

The excessive use of antibiotic drugs causes severe water pollution and poses a serious threat to the ecosystem and human health, even at a low concentration [161]. Thus, the adsorbents capable of removing antibiotic pollutants from water have been topics of concern in recent years [37,40,162,163,164,165,166]. Natural plant gums-based adsorption materials are promising and have potential to be used for the removal of antibiotic pollutants. Thakur et al. [167] prepared a xanthan gum-cl-poly(itaconic acid)/bentonite composite hydrogel via the grafting polymerization reaction between itaconic acid and xanthan gum. The hydrogel adsorbent can efficiently adsorb ampicillin, with a saturation adsorption capacity of 245 mg/g (initial concentration: 100 mg/L; adsorbent dose: 0.4 g/L). The number of functional groups on the adsorbent increased after the grafting polymerization reaction, and the electrostatic and hydrogen bonding attraction of the adsorbent towards ampicillin was also enhanced, resulting in a significant improvement in the adsorption capability (Figure 6).

Bisphenol A is a toxic monomer used for the production of polycarbonate plastics and epoxy resin. Bisphenol A may induce certain diseases even at a low concentration (1 mg/m^3^), such as hypertension, cardiovascular disease and metabolic syndrome. Therefore, the removal of Bisphenol A from water by using simple and efficient materials or technologies is highly desired. Owing to their high adsorption capacity and fast adsorption rate, natural polymers-based hydrogels have shown their advantages for the decontamination of Bisphenol A pollutants. Chen et al. [168] synthesized a temperature-sensitive hydrogel (xanthan gum-graft-N-isopropylacrylamide) in an aqueous medium using citric acid as a crosslinking agent. The hydrogel can absorb Bisphenol A with a high adsorption capacity of 458 mg/g (initial concentration: 800 mg/L; adsorbent dose: 0.4 g/L), showing a great potential for the decontamination of Bisphenol A in wastewater.

### 3.3. Pectin-Derived Adsorbents 

Pectin is a natural polysaccharide produced from the by-product of apple or citrus peel-α-D-galactouronic acid residues, which is composed of at least 22 different glycosidic bonds and 12 types of neutral sugar branching [169]. The main functional groups on pectin are hydrophilic electron-rich groups, such as hydroxyl, carboxyl and acylamino groups [170,171,172]. Pectin can complex with multivalent metal ions to form an ion-crosslinked network gel [173]. Pectin is negatively charged, and can form electrostatic attraction with positively charged ions. Pectin has good hydrophilicity, similar to cellulose, chitosan and sodium alginate, and can be used to prepare environmentally friendly hydrophilic adsorbents [174,175]. At present, pectin-based adsorbents can be prepared by modification with reactive molecules [176], direct composite with other components, and the graft polymerization reaction, which can be used for the removal of heavy metal ions, dyes, antibiotics, and other pollutants.

#### 3.3.1. Application for Capturing Heavy Metal Ions

**Figure 7:**
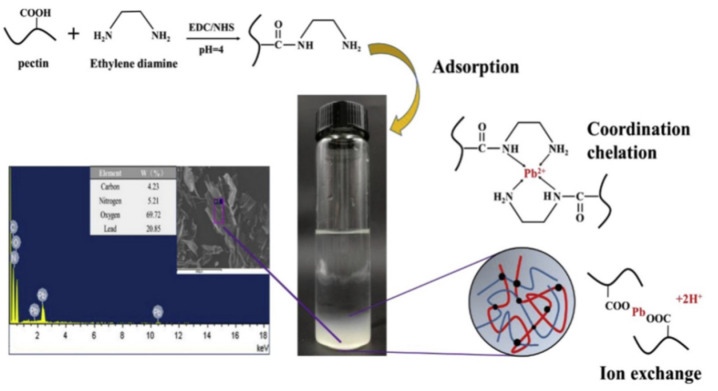
The process of ethylenediamine-modified pectin preparation and its adsorption mechanism for Pb^2+^ [177].

Pectin-based adsorbents show better application prospects for the removal of heavy metal ions, due to their excellent ability to complex to metal ions and their eco-friendly advantages. The abundant carboxyl groups on pectin enables it to be directly used for the adsorption of metal ions. Arachchige et al. [176] evaluated the adsorption properties of the modified sweet potato pectin in removing Pb(II) from water, and proved that the modified sweet potato pectin is highly efficient in adsorbing Pb(II) ions, with an adsorption capacity of 263.15 mg/g. The excellent adsorption ability is ascribed to the synergistic effect of the chemical complexation of O-containing groups (such as -O-H, -COO-) towards Pb(II) ions, ion exchange and electrostatic interaction. Liang et al. [177] prepared an ethylenediamine-modified pectin with different amidation degrees. The adsorbent shows an excellent adsorption efficiency for Pb(II) ions (≥94%) due to the contribution of ion exchange, and the chemical chelation action of carboxyl and the amino groups (Figure 7). Because pectin contains carboxyl groups capable of capturing metal ions, it can be directly used to prepare adsorbents with an ideal adsorption capability. Martins et al. [178] prepared a persistent chitosan/pectin (O-methoxylation degree: 56%) blending film with a pectin content of 74% through a solvent evaporation method. The blending film shows an adsorption capacity of 29.20 mg/g for Cu(II) ions (initial concentration: 100 mg/L). Wang et al. [179] prepared a pectin/activated carbon microsphere via a simple gel method, without chemical cross-linking. The microsphere shows a high adsorption capacity of 279.33 mg/g for Pb(II) (initial concentration: 300 mg/L; adsorbent dose: 1 g/L) and excellent reusability (above 95.5% of adsorption efficiency remained after reused 10 times). The ion exchange between Pb(II) and Ca(II), electrostatic adsorption, chemical complexation, and physical adsorption mainly contribute to the adsorption. Incorporating inorganic components into the pectin matrix can also improve its comprehensive performance. Kusrini et al. [180] prepared a pectin/activated carbon composite by an impregnation method, using pectin extracted from banana agricultural waste as raw material. The composite adsorbent shows the adsorption capacities of 21.80 mg/g, 27.78 mg/g, 18.22 mg/g, 21.09 mg/g and 24.91 mg/g for La(III), Y(III), Nd(III), Sm(III) and Ce(III), respectively. Badura et al. [181] prepared a spherical hybrid adsorbent with an excellent removal rate of cesium ions (100%) (initial concentration: 115 mg/L; adsorbent dose: 3 g/L) by incorporating Prussian blue into cross-linked pectin. Bok-Badura et al. [182] prepared an adsorbent composed of pectin and nanoscale titanium dioxide. The adsorbent shows saturation adsorption capacities of 87.68, 76.16, 33.15 and 171.98 mg/g for Cu(II), Cd(II), Zn(II) and Pb(II), respectively. Pectin-based adsorbents can also be prepared into films that can be easily recovered after use. Hastuti et al. [183] prepared an adsorption film by pouring a mixture solution of chitosan and pectin into a polypropylene container, and then drying it. The pectin film can adsorb Pb(II) with a high removal rate of 92.68%, and shows excellent stability and acid resistance.

**Figure 8:**
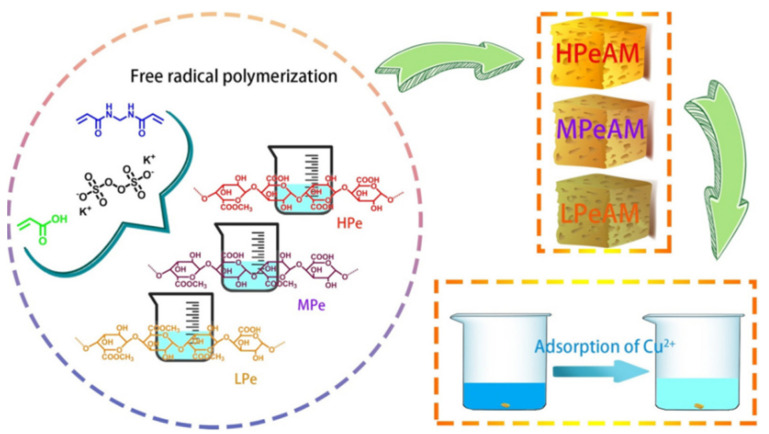
The preparation process of pectin-based adsorbent by a grafting polymerization reaction and its adsorption process for Cu(II) [184].

Figure 7 shows that pectin-based adsorbents may adsorb heavy metal ions, mainly via a chemical complexation interaction, so introducing more functional groups is certainly helpful to improve the adsorption capacity. Shen et al. [184] prepared a pectin-*graft*-poly(acrylic acid) hydrogel via a free radical polymerization route, using modified pectin with different esterification degrees as the raw material. The increase in the number of carboxyl groups may strengthen the adsorption driving force for the adsorption of heavy metal ions, such as Cu(II) (Figure 8).

#### 3.3.2. Application for Capturing Dyes

Due to the toxicity and chromogenicity of synthetic dyes, the water pollution caused by dye brings severe harm to the environment and people’s health. Therefore, people pay more attention to developing environmentally friendly materials from natural polymers to eliminate the dye pollutants [48]. Pectin becomes a potential candidate for the development of purification materials for pollutants. Pectin can be crosslinked by Ca^2+^ ions to form an insoluble gel adsorbent. Li et al. [185] prepared a non-toxic pectin-based adsorbent with a high adsorption capacity of 354.6 mg/g (initial concentration: 1100 mg/L) for methylene blue, via an ionic crosslinking method with Ca^2+^ as a crosslinking agent. Shakibi et al. [186] also synthesized a pectin-based hydrogel crosslinked with Ca^2+^ ions on the polyester fabrics. The fabric treated with chitosan/pectin composite hydrogel had a higher dye adsorption capability than those treated with pectin and cationic surfactant. Khorasani et al. [187] prepared a magnetic Fe_3_O_4_/pectin/chlorella vulgaris (1:1) blending composite adsorbent without any chemical reaction. The incorporation of Fe_3_O_4_ particles not only makes the composite adsorbent easy to separate, but also may enhance the adsorption capacity towards malachite green dye (adsorption capacity: 197.5 mg/g) (adsorbent dose: 0.5 g/L; initial concentration: 100 mg/L); it can also be recycled for multiple cycles. The pectin-based adsorbents can be made into special shapes, such as membranes or sponges. Hastuti et al. [188] prepared a pectin-based adsorption film capable of adsorbing methylene blue dye by casting the solution of pectin into a 5% acetic acid solution. The pectin film with active carboxyl and hydroxyl groups on it can capture positively charged methylene blue with an adsorption capacity of 19.39 mg/g. Attallah et al. [189] prepared a magnetic pectin/chitosan sponge capable of adsorbing methylene blue dye from water by a shrinking gel method. It was calculated, by the Sips equation, that the maximum adsorption capacity of the sponge towards methylene blue is 174 mg/g. The pectin-based adsorbent with a specific shape has the advantages of convenient use, easy recovery and disposal, and will receive increasing attention in the future.

#### 3.3.3. Application for Capturing Antibiotics and Other Organic Pollutants

The removal of emerging pharmaceuticals or phenolic pollutants from water by efficient materials or methods is desirable but challenging. The development of environmentally friendly adsorption materials, capable of eliminating pharmaceutical pollutants, is always a concern. Kadam et al. [190] prepared a magnetic composite adsorbent (Fe_3_O_4_@Pec-OPB) using pectin extracted from the waste biomass of orange peel (Pec-OPB) as the raw material. The adsorbent was proved to have potential for purifying drug-containing wastewater, with a high adsorption capacity of 120 mg/g for sulfamethoxazole. Tanasale et al. [191] prepared an adsorbent using the pectin separated from orange peel as the raw material. Under the optimal conditions (contact time: 20 min; pH 3.0; phenol concentration: 25 ppm), the adsorbent can efficiently adsorb phenol with a removal rate of 80.97%. Although natural plant gums-based adsorbents show great prospects in the removal of organic pollutants, such as pharmaceuticals and phenols, the related research is rare; they deserve to be paid more attention with the increasing attention paid to emerging pollutants.

### 3.4. Carrageenan Gum-Derived Adsorbents 

**Figure 9:**
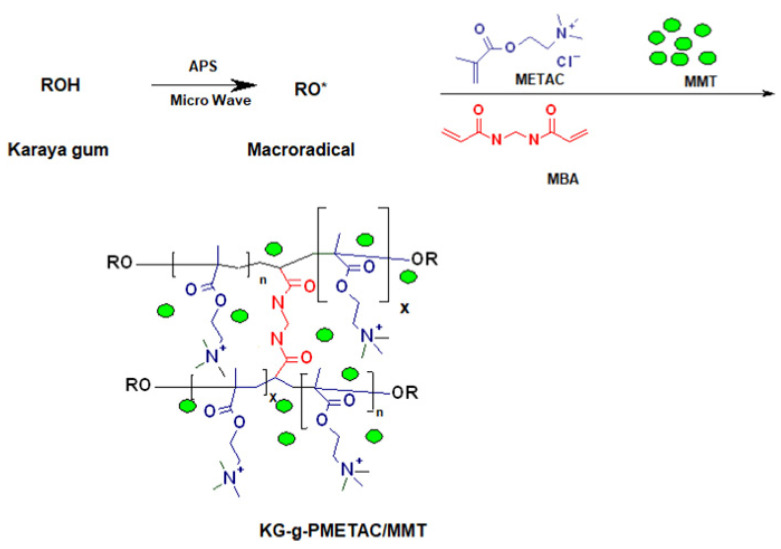
Formation process of KG-g-PMETAC/MMT composite adsorbent [198].

Carrageenan gum is a hydrophilic natural polymer extracted from red algae seaweed. It is composed of sulfated or non-sulfated galactose and 3,6-dehydrated galactose connected alternately by α-1,3-glycosidic bond and β-1,4 bond. Carrageenan gum has been widely used in biology, chemistry, food science and technology, medical treatment and other fields as a gelling agent, thickener, deformation agent or stabilizer; this is due to its biodegradability, biocompatibility and high viscosity [192,193]. The carrageenan gum is easily modified to form derivatives or new materials with improved performance. At present, carrageenan gum can directly compound with other components to prepare granular, membrane, bead, or aerogel-shaped adsorbents [194,195,196,197], and can also be used to prepare adsorbents with a three-dimensional network structure via a graft polymerization reaction [198]. The carrageenan gum-graft-poly((2-methacryloyloxy ethyl) trimethyl ammonium chloride)/montmorillonite (KG-g-PMETAC/MMT) polymer composite adsorbent can be prepared via a microwave-assisted graft polymerization reaction route (Figure 9). In the presence of an initiator, karaya gum may react with monomers to form a grafting polymer adsorbent. The montmorillonite can be embedded in the polymer matrix to form a nanocomposite; these are not only eco-friendly, but also highly efficient in adsorbing heavy metals, dyes, antibiotics and other pollutants.

#### 3.4.1. Application for Capturing Heavy Metal Ions

**Figure 10:**
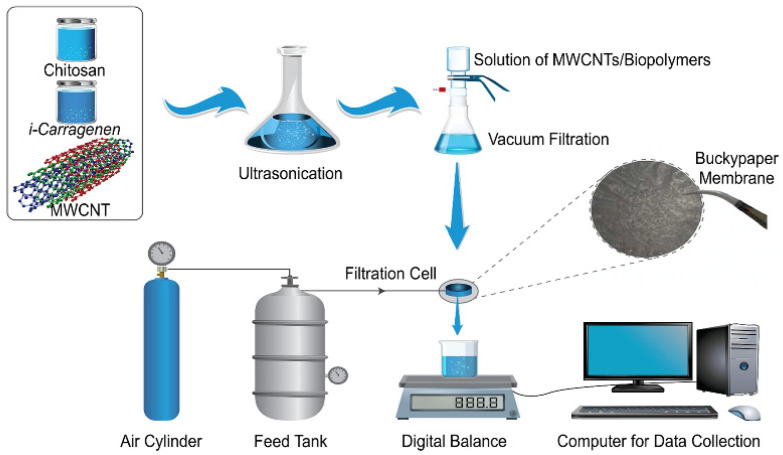
Preparation process of chitosan/carrageenan gum/carbon nanotube composite membrane [195].

The adsorbents derived from natural plant gums, such as carrageenan gum, have attracted much attention for their ability to remove heavy metals from water. Many studies reported the preparation process of adsorption materials by incorporating diversified components into the carrageenan gum matrix. Zhuang et al. [194] prepared a carrageenan gum/graphene oxide hybrid hydrogel with an excellent adsorption capacity of 331 mg/g for Hg(II) (initial concentration: 100 mg/L; adsorbent dose: 3 g/L). In addition, the hydrogel can adsorb simultaneously Hg(II) and methylene blue in water. Wang et al. [199] prepared a polyethylenemine/κ-carrageenan composite using κ-carrageenan the as raw material. The synergistic effect of polyethyleneimine and carrageenan gum in the composite contributes to removing Cu(II), with a high removal rate of 98%. The adsorbents can be made into magnetic particles, membranes, beads or aerogels, that are easily recovered after use. Rahmani et al. [200] synthesized a magnetic adsorbent with high stability, rapid recovery performance and excellent adsorption capacity (114.6, 107.6, 99.2, and 94.2 mg/g for Pb(II), Cu(II), Hg(II) and Cd(II), respectively (initial concentration: 200 mg/L) using carrageenan gum, nitrogen doped carbon dots and Fe_3_O_4_ as the raw materials. The adsorbent also maintains a high removal efficiency (88%-98%) for metal ions in actual wastewater, and can be recovered by an external magnetic field. Alshahrani et al. [195] prepared a multi-walled carbon nanotubes (MWCNTs)/chitosan/carrageenan gum composite membrane via a vacuum filtration technology (Figure 10) by using carrageenan gum, chitosan and MWCNTs as raw materials. The composite membrane shows excellent thermal stability, mechanical properties (tensile strength: 30.69 ± 2.6 MPa) and adsorption efficiency for heavy metal ions (removal rate: 38%, 41%, 92%, 65%, 66% and 91% for Co(II), Ni(II), Cu(II), Cd(II), Ba(II) and Pb(II), respectively). The composite membrane possesses potential for the decontamination of heavy metals from wastewater. Ali et al. [201] prepared magnetic biopolymer-based beads using carrageenan gum and different types of cellulose as the raw materials. With the increase in the content of carrageenan gum in the adsorbent, the content of carboxyl groups also increased, and the removal rates of the adsorbent for Cu(II), Pb(II), Ca(II), Mg(II) and Fe(II) also increased to 90%, 65%, 25%, 56% and 55%, respectively. The magnetic beads can be directly recycled by a simple sieving process after use. Abdellatif et al. [202] prepared a magnetic aerogel by physically crosslinking three components: magnetic nanoparticles, carrageenan gum and a polyamide amide dendrimer. The adsorbent can adsorb Cr(VI), Co(II), Cu(II), Cd(II) and Mn(VII), and the removal rate of Co(II) reaches 99%. The aerogel adsorbent is easily recovered after use. Carrageenan gum can also be used as a precursor to prepare the carbon-based adsorption materials. Zhang et al. [203] synthesized, in situ, an iron sulfide (FeS)/carbon fiber composite adsorbent using carrageenan gum as a precursor. The FeS/carbon fiber composite shows a high removal capacity, of 81.62 mg/g for Cr(VI). The reduction in FeS, the electrostatic adsorption of carbon fiber, and a Cr(III)-Fe(III) complexation reaction mainly contribute to the removal of Cr(VI).

The crosslinking or graft polymerization reaction of carrageenan gum with other components may yield more adsorbents containing functional groups. Abdellatif et al. [204] prepared a i-carrageenan aerogel with a hierarchical macroporous cellular structure, via the crosslinking reaction between carrageenan gum and a polyamide amide hyperbranched polymer. The sample with the higher nitrogen content has the highest adsorption efficiency for the metal ions Cr(VI), Mn(II), Co(II), Cu(II) or Cd(II). 

#### 3.4.2. Application for Capturing Dyes

Carrageenan gum-derived adsorption materials have been used in the decontamination of dye wastewater [205]. The materials with excellent dye adsorption properties can be obtained by directly compounding carrageenan gum with other components. Duman et al. [206] prepared a magnetic carrageenan gum/oxidized multi-walled carbon nanotubes/iron oxide (Fe_3_O_4_) adsorbent with adsorption capacities of 0.116 mmol/g for cationic dye crystal violet and 0.0185 mmol/g for anionic dye reactive black 5. The adsorbent can be recovered by an external magnetic field after use. Vaid et al. [207] prepared a 3D network hydrogel, crosslinked with kappa carrageenan and tamarind kernel powder. The hydrogel shows the maximum adsorption capacity of 840.33 mg/g for cationic dye BG and 168 mg/g for anionic dye RB, respectively (adsorbent dose: 1g/L). The good shape-forming performance of carrageenan gum means it can be prepared into recyclable adsorbents, such as film, beads, and aerogels. Ulu et al. [208] prepared a recyclable environment-friendly chitosan/κ-Carrageenan/acid activated bentonite composite membrane. The addition of bentonite in the membrane enhanced its mechanical strength and hydrophobicity. The removal rate of methylene blue dye by the membrane was up to 98% (adsorbent dose: 0.05 g; initial concentration: 50 mg/L). Reyaz et al. [196] synthesized Cu(II)-crosslinked graphene oxide/alginate/carrageenan gum composite hydrogel beads with an adsorption capacity of 132 mg/g for methylene blue (initial concentration: 260 mg/L; adsorbent dose: 1 g/L). The mechanical properties of the hydrogel beads can be adjusted by changing the concentration of copper ions, which is favorable for the practical application of the membrane. Song et al. [197] prepared a graphene oxide/ι- Carrageenan composite aerogel with an adsorption capacity of 245.28 mg/g for methylene blue in solution. The aerogel adsorbent can be easily recovered and reused for multiple cycles, and can reach a removal rate of 91.9% after being reused three times. Because the aerogel is negatively charged, it can selectively adsorb positively charged methylene blue dye from the methylene blue/methyl orange binary mixed system.

The comprehensive performance of carrageenan gum-based adsorbents can be further improved by introducing more functional groups. Preetha et al. [198] prepared a polymer composite (KG-g-PMETAC/MMT) by a graft polymerization reaction between carrageenan gum and (2-methacryloyloxy ethyl) trimethyl ammonium chloride in the presence of montmorillonite, through a microwave assisted synthesis route. The hydrophilic functional groups in the adsorbent, such as COOH, -OH and -N^+^(CH_3_)_3_, make it expandable and capable of complexing more metal ions; therefore, the adsorption capacity and rate can be significantly improved. The maximum adsorption capacity of the adsorbent reaches 155.85, 149.64, 128.78 and 137.77 mg/g for methylene blue, toluidine blue, crystal violet and sky blue B, respectively (adsorbent dose: 0.5 g/L; initial concentration: 100 mg/L). These results show that carrageenan gum-based adsorbents have great potential for the decontamination of dye wastewater; indeed, the development of a new generation of superb adsorption materials based on carrageenan gum is urgently needed.

#### 3.4.3. Application for Capturing Pharmaceuticals

The drug pollutants in wastewater may cause serious harm to the environment and human health. In order to eliminate the harm of pharmaceuticals, the low-density lanthanum-modified κ-carrageenan gum/sodium alginate aerogel was synthesized by a simple sol-gel method [209]. The aerogel can efficiently adsorb ciprofloxacin hydrochloride in water with an adsorption capacity of 179.97 mg/g (initial concentration: 300 mg/L; adsorbent dose: 0.32 g/L), even in real water. The adsorption of ciprofloxacin hydrochloride by the aerogel was mainly driven by electrostatic interaction, a π-π electron donor-receptor interaction, a hydrogen bond, and hydrophobic interaction. Li et al. [210] prepared a recyclable carboxymethyl cellulose/κ-carrageenin gum composite with an interpenetrating network structure and high adsorption capacity for antibiotics ciprofloxacin (1.271 mmol/g). Myrsini et al. [211] synthesized an isocyanate modified kappa-carrageenan gum (k-CAR) polymer for the adsorption of anti-inflammatory drugs, such as the antiepileptic compounds carbamazepine and diclofenac acid. The adsorption capacity of the adsorbent is 7.07~13.78 mg/g for carbamazepine and 22.66~49.29 mg/g for diclofenac acid. Nanaki et al. [212] synthesized a green adsorbent by crosslinking carrageenan gum with glutaraldehyde. The adsorbent has a low swelling degree and a better adsorption capability towards metoprolol in an aqueous solution (adsorption capacity: 109 mg/g at 20 °C and 178 mg/g at 40 °C). The adsorbents that are suitable for the adsorption of drug pollutants can be prepared by the composite of carrageenan gum and other components. Soares et al. [213] synthesized biological adsorbent carrageenan-cladding Fe_3_O_4_ nanoparticles with hybrid siliceous shell (Fe_3_O_4_@SiO_2_/SiCRG), which have a high surface volume ratio and an excellent adsorption capacity for metoprolol (447 mg/g). The electrostatic interaction between carrageenan gum and metoprolol is the main driving force for adsorption.

In addition, carrageenan gum can serve as a carbon source to prepare activated carbon adsorbent with a better adsorption efficiency for drug pollutants. Nogueira et al. [214] prepared porous activated carbons (AC-κ, AC-ι and AC-λ, respectively) with large specific surface areas (up to 2800 m^2^/g) using κ-, ι- and λ-carrageenan gum as carbon sources. The carbons can rapidly adsorb ciprofloxacin in water, withthe high adsorption capacities of 330 mg/g, 422 mg/g and 459 mg/g for AC-ι, AC-κ, and AC-λ within 5 min, respectively. The removal rate of ciprofloxacin by all activated carbons is above 99%. 

#### 3.4.4. Application for Capturing Other Organic Pollutants

Carrageenan gum-derived adsorption materials have also been applied for the removal of herbicides and other organic pollutants. Dibromamine is one of the most widely used cationic quaternary ammonium herbicides, which can cause severe harm to aquatic organisms and human health. In order to remove these pollutants, Duman et al. [215] developed a magnetic oxidized multi walled carbon nanotubes (OMWCNT)-k-carrageenan-Fe_3_O_4_ adsorbent using carrageenan gum, OMWCNT and ferric oxide as starting materials for the adsorption of toxic quinquium dibromide. The result shows that the chemical adsorption process is dominant for capturing quinquium dibromide, and the maximum adsorption capacity is 10.7 mg/g. Huang et al. [216] synthesized the physically crosslinked double network hydrogel adsorbent carrageenan gum/chitosan/calcium ion for removal of 3-Nitro-1,2,4-triazole-5-one (NTO) from water. The adsorbent is capable of adsorbing 73 mg/g of NTO via a hydrogen bonding interaction, and the adsorbed NTO can be desorbed using NaOH solution as a desorbent to recover the adsorbent and NTO.

### 3.5. Adsorbents Derived from Arabic Gum 

**Figure 11:**
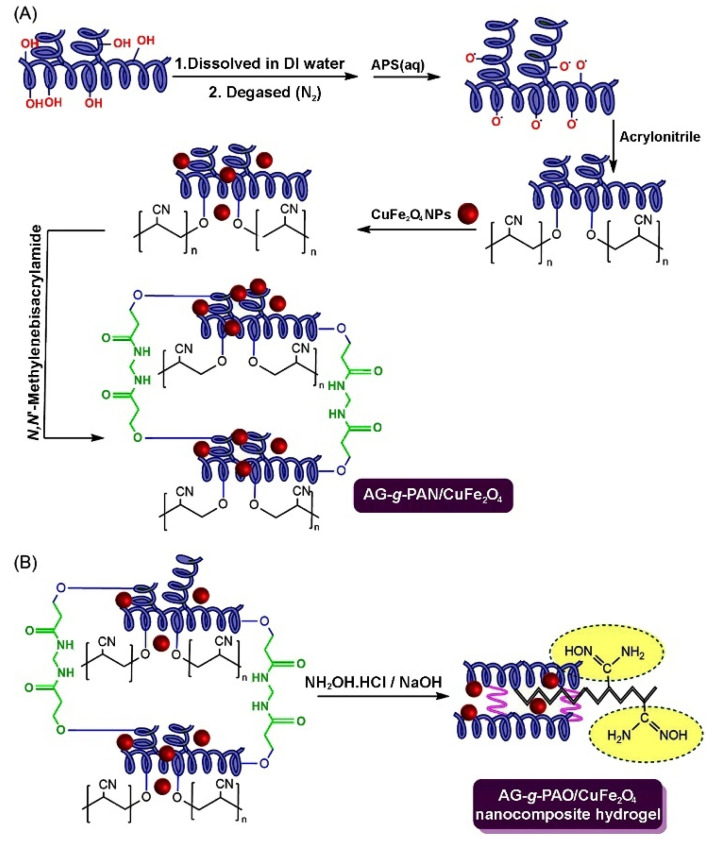
(**A**,**B**) The grafting polymerization reaction process of polyacrylonitrile onto arabic gum and amidoximation reaction [217].

Arabic gum is a natural polymer; it is a complex, highly branched macromolecule composed of β-D-galactopyranosyl units, connected by 1,3-linkage. The reactive hydroxyl groups of arabic gum make it easily cross-linked or grafted to form adsorption materials with multiple active groups. In addition, arabic gum can compound with other components to form a composite adsorbent. Therefore, arabic gum-based adsorbents can be prepared by the direct composite or graft polymerization routes. A variety of grafting copolymers, with improved performance, can be prepared by a free radical graft polymerization reaction among the initiator, crosslinking agent, monomer, and other components in the aqueous solution. The general graft polymerization reaction process is shown in Figure 11 [217]. In the initiation stage, the initiator (such as persulfate) can be excited by heat or reductant to generate free radicals. The radicals may further attack the active groups on the macromolecular chains of arabic gum and the monomers to generate more free radicals. In the stage of chain propagation, the monomers with single electrons are quickly polymerized or grafted onto the macromolecular chains and these chains are crosslinked with each other to form a crosslinked graft polymer network. Based on this reaction principle, the arabic gum-graft-polyamidoxime/CuFe_2_O_4_ nanocomposite hydrogel was prepared via the polymerization reaction among arabic gum, the initiator, crosslinker, monomers and inorganic components, and the subsequent amidoximation reaction. The adsorbent shows a high adsorption capacity of 769.23 mg/g for the pesticide chloropyrifos [217].

#### 3.5.1. Application for Capturing Heavy Metal Ions

Arabic gum-based adsorbents provide a new approach for removing heavy metal pollutants. Banerjee et al. [218] developed a magnetic adsorbent composed of arabic gum and Fe_3_O_4_ nanoparticles. Because arabic gum is coated on the outer surface of the adsorbent, the diffusion rate of Cu(II) ions into the adsorbent is fast, and the adsorption process rapidly reaches equilibrium within 2 min (maximum adsorption amount: 38.5 mg/g). The Cu(II) adsorbed on the adsorbent can be desorbed with HCl solution (pH 1.5), and the desorption efficiency reaches 93%. After three regenerations, the adsorption performance of the adsorbent does not decrease obviously. Nowroozi et al. [219] prepared a magnetic MgFe_2_O_4_/arabic gum complex via a simple ultrasonic assisted precipitation route, and further modified it with l-cysteine to form a modified magnetic arabic gum adsorbent. The adsorbent can efficiently adsorb Hg(II) (adsorption capacity: 250 mg/g), and can be recycled more than three times after use. Besides toxic heavy metal ions, radioactive waste is harmful to most life forms. To eliminate the pollution of radioactive metal ions, Mahmoud et al. [220] prepared a magnetic arabic gum/polyacrylamide/graphene [(GA/PAAM/GR)-MNC] and magnetic arabic gum/polyacrylamide/silica [(GA/PAAM/SiO_2_)-MNC] nanocomposites. The (GA/PAAM/GR)-MNC and (GA/PAAM/SiO_2_)-MNC nanocomposites show a high removal efficiency of 89% and 92% for Cs^+^ and 97% and 95.5% for Co(II), respectively.

Introducing more functional groups onto the macromolecular chains of arabic gum is an effective way to improve its complexing ability toward heavy metals. Elbedwehy et al. [221] prepared an arabic gum-graft-polyacrylonitrile adsorbent with a cross-linking network structure. The adsorption efficiency of the adsorbent for heavy metal ions reached 93% (adsorption capacity: 150 mg/g) within 2 min, and the maximum adsorption capacity reached 1017, 413 and 396 mg/g for Pb(II), Cd(II) and Cu(II), respectively. The adsorption efficiency remains at 99% after being reused three times. After treatment with 0.2 M HNO_3_ solution, Pb(II), Cd(II) and Cu(II) can be desorbed almost completely (desorption rate: 96%, 99% and 99% for Pb(II), Cd(II) and Cu(II), respectively). The microwave-assisted technology can be used to synthesize an arabic gum-g-poly (3-chloro-2-hydroxypropylmethacrylate)/Fe_3_O_4_ nanocomposite hydrogel for capturing metal ions from aqueous solutions [222]. The introduction of magnetic nanoparticles is conducive to improving the pore structure of hydrogel, thus improving the adsorption capacity of the adsorbent for Cu(II) (307.5 mg/g) and Hg(II) (292.8 mg/g). This proves that the arabic gum-grafted polymer has a great application potential for the decontamination of heavy metal ions.

#### 3.5.2. Application for Capturing Dyes

The plentiful functional groups on the macromolecular chain of arabic gum makes it capable of forming hydrogen bonding or electrostatic interactions with other organic molecules, such as dyes, to enhance its adsorption properties. Therefore, arabic gum-derived adsorbents possess potential to be used for the removal of dye pollutants. The arabic gum-modified magnetic nanoparticles can remove 96.3% of Congo red in 100 mg/L solution [223]. An arabic gum-capped polyaniline nanocomposite hydrogel was prepared by a dispersion polymerization process. The optimal adsorbent shows an adsorption capacity of 35.41 mg/g and a removal rate of 89% for methylene blue (initial concentration: 10 mg/L) [224]. The adsorption performance can be improved further by introducing more functional groups through a grafting polymerization reaction. Ibrahim et al. [225] prepared a poly(acrylic amide-co-3-Allyloxy-2-hydroxy-1-propanesulfonic acid sodium salt)/arabic glum semi-IPN hydrogel, which can adsorb dye with a high adsorption capacity of 655.2 mg/g; the adsorption capacity remains high after being reused for more than 5 cycles. Sharma et al. [226] prepared an arabic gum-cl-poly(acrylamide) nanohydrogel, capable of efficiently adsorbing the toxic crystal violet dye from water (adsorption capacity: 90.90 mg/g; removal rate: 79% for dye in 100 mg/L of solution).

#### 3.5.3. Application for Capturing Other Organic Pollutants

Arabic gum-based adsorption materials can provide a solution to eliminating pharmaceutical pollutants. Darvishi et al. [227] prepared a hydrogel adsorbent by cross-linking pectin and O-carboxymethyl chitosan using oxidized arabic gum as a cross-linking agent. The adsorbents can efficiently adsorb antibiosis levofloxacin and delafloxacin with the adsorption capacities of 147.6 mg/g (for delafloxacin) and 155.5 mg/g (for levofloxacin), respectively in acidic medium (pH 3.9). Mota et al. [228] synthesized an arabic gum-graft-poly(acrylic acid) hydrogel by a free radical graft polymerization reaction with an adsorption capacity of 15.16 mg/g (at 25 °C) for diazepam in water. The adsorbent maintains a good adsorption capacity, even after being reused five times. With the increasing concern regarding the pollution caused by pharmaceuticals, more attention should be paid to fabricate environmentally friendly adsorbents for the removal of antibiotics and other types of pharmaceuticals.

### 3.6. Welan Gum-Derived Adsorbents

Welan gum is a heteropolysaccharide produced from aerobic fermentation using bacteria of the genus Alcaligenes. It is composed of repeated tetrasaccharide units of D-glucose, D-glucuronic acid, D-glucose and L-rhamnose. The presence of D-glucuronic acid in welan gum produces polyelectrolyte characteristics, good biocompatibility and biodegradability [229]. Welan gum is widely used in petroleum, construction, medicine, food and other fields, due to its excellent viscosity and stability at high temperatures [230,231]. The application of welan gum in purifying pollutants is still in its infancy, and more research is needed to develop highly efficient welan gum-based adsorbents.

Welan gum-based adsorbents have shown great advantages for the removal of heavy metals and dye pollutants. In order to remove heavy metal ions in water, Liu et al. [232] prepared a welan gum-modified cellulose adsorbent with the maximum adsorption capacity of 83.6, 77.0 and 67.4 mg/g for Cd(II), Pb(II) and Cu(II) ions, respectively. In addition, the welan gum-based adsorbents were also used to purify dye wastewater. Yu et al. [233] developed an environmentally friendly welan gum/graphene oxide nanocomposite hydrogel with an excellent capability to adsorb water-soluble dyes, such as methylene blue, methyl violet, amide black 10B, rhodamine 6G and chrome azurol S. Jiao et al. [234] prepared a welan gum/graphene oxide hydrogel and studied its adsorption properties for the anionic dye chrome azurol. It was found that the adsorption capacity of the hydrogel is pH-dependent, and the acidic conditions are more favorable for the adsorption of dye. Deng et al. [235] modified the cellulose microsphere coated with emulsified carbon nanotubes with welan gum to prepare a composite adsorbent with a maximum adsorption capacity of 302.1 mg/g (adsorbent dosage: 0.4 g/L; dye concentration: 180 mg/L) for methylene blue. This demonstrates that the welan gum possesses great potential to be used for the development of eco-friendly and superb dye adsorbents.

### 3.7. Gellan Gum-Derived Adsorbents 

**Figure 12:**
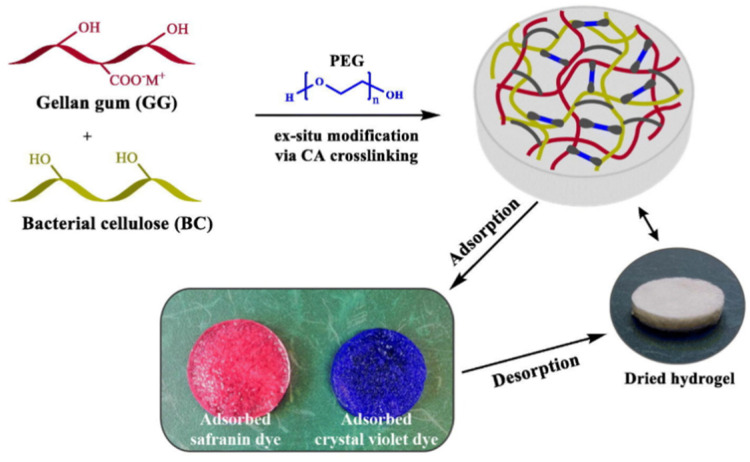
The main interaction for the adsorption of dyes by gellan gum-based adsorbent and the state of adsorbent before and after dye adsorption [237].

Gellan gum is a linear, water-soluble anionic polymer, which is composed of tetrasaccharides with a 2:1:1 molar ratio of D-glucose, D-glucuronic acid and L-rhamnose. Gellan gum can compound with other polymers or particles, and can react with vinyl monomers to improve its performance and functionality. Gellan gum-derived adsorbents exhibit better prospects in the removal of heavy metals, dyes and other pollutants. Wang et al. [236] prepared a magnetic gellan gum/Fe_3_O_4_ composite adsorbent which can adsorb heavy metal ions by an ion-exchange process and a complexing action of carboxyl groups, and can be separated by a magnetic field after use. The capture capacity of the adsorbent toward different types of metal ions is in the order of Pb(II) (260.13 mg/g) > Cr(III) (240.54 mg/g) > Mn(II) (127.63 mg/g). Nguyen et al. [237] prepared an adsorbent with an excellent mechanical strength and adsorption performance for dye by a cross-linking reaction among gellan gum, bacterial cellulose and citric acid (as crosslinker). The adsorbent can adsorb dyes by hydrogen bonding and electrostatic interaction (Figure 12), and the adsorption capacity for safranin die and crystal violet are 17.57 mg/g and 13.49 mg/g, respectively. Shabani and Dinari [238] prepared a modified Cu-Ca-Al double hydroxide/polymer matrix nanocomposite adsorbent by a simple synthesis route, and then the resultant material was incorporated into gellan gum to form nanocomposite adsorbents with different amounts of filler. Under the action of an electrostatic and hydrogen bond interaction, the nanocomposite adsorbent can efficiently adsorb Congo red dye, with an adsorption capacity of 99.9 mg/g (initial dye concentration: 300 mg/L; adsorbent amount: 2 g/L). In view of the disadvantage of traditional adsorbents that are not easy to recover, the blocky aerogels that can be easily recovered after are welcome. Dong et al. [239] prepared a sodium alginate/gellan gum aerogel by blending gellan gum with sodium alginate. The aerogel has good chemical stability and an excellent adsorption capacity (1456.45 mg/g) for methylene blue dye. After five adsorption-desorption cycles, the adsorption capacity of the aerogel only decreases by 1.44%, which indicates that the adsorbent with pure natural components has great application prospects as a green adsorbent. Sukumar et al. [240] prepared a gellan gum/MgO composite adsorbent with a better adsorption ability towards cationic dye malachite green, with the highest single-layer adsorption capacity of 92.5 mg/g.

### 3.8. Tara Gum-Derived Adsorbents 

**Figure 13:**
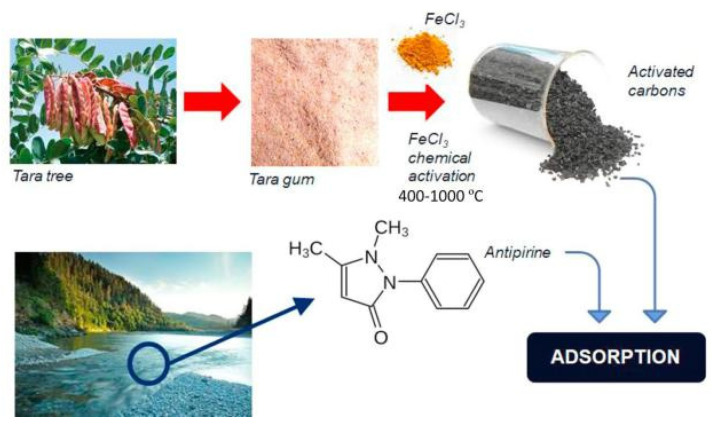
Preparation process of Tara gum-derived activated carbon and its application for adsorption antipyrine in water [242].

Tara gum is a natural plant gum extracted from Caesalpinia spinosa (Molina), a native Peruvian shrub. It has good biocompatibility and safety. Compared with other polysaccharides, such as cellulose and chitosan, Tara gum has a relatively low molecular weight. Therefore, Tara gum is independently used for the development of product with difficulty, which usually blends with other polysaccharides to develop various materials. In recent years, Tara gum was found to possess great potential for the preparation of adsorption materials for removal in industrial pollutants, such as dyes. Gomez-Maldonado et al. [241] prepared composite adsorption beads by blending the solution of Tara gum in urea/sodium hydroxide alkaline medium with lignocellulose nanofibers. The beads show an adsorption capacity (13.7 mg/g) for methylene blue better than pure cellulose beads. Tara gum-derived adsorption materials also show good adsorption performance for other types of organic pollutants. Bedia et al. [242] prepared an activated carbon adsorbent via the activation of Tara gum with ferric chloride (Figure 13). The Fe3+ may promote the carbonization of Tara gum to form better activated carbon. The adsorbent has a high specific surface area (1680 m^2^/g) and excellent adsorption capacity of 275 mg/g for antipyrine (initial concentration: 100 mg/L; adsorbent dose: 0.2 g/L).

### 3.9. Gum Tragacanth-Derived Adsorbents 

**Figure 14:**
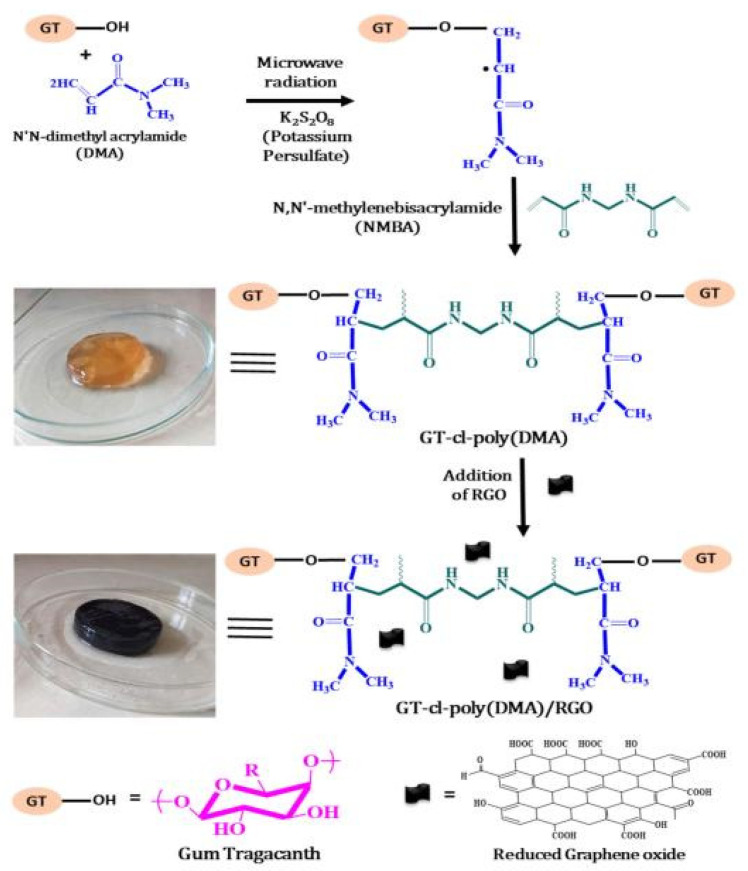
A scheme illustrated the preparation process of gum tragacanth-cl-N,N-dimethylacrylamide/graphene hydrogel composite [244].

Gum tragacanth is a complex, heterogeneous, and hydrophilic natural carbohydrate polymer, which is composed of a water-soluble astragalin with a high molecular weight and water-soluble basolin. Due to its biocompatibility, gum tragacanth has shown significant potential in biomedical applications and hydrogel membranes. In recent years, with the increasing demand for environmentally friendly adsorbents in the decontamination of wastewater, the adsorbents derived from gum tragacanth have been of concern for the removal of heavy metals, dyes, or oily pollutants. In order to treat wastewater containing heavy metals, Mallakpour et al. [243] prepared an environmentally friendly nanocomposite hydrogel adsorbent by compounding gum tragacanth and ethylene diamine tetra-acetic acid-modified CaCO_3_ nanoparticles, which shows a high removal rate of 87% for Pb(II) ions in solution (adsorption capacity: 70 mg/L) (adsorbent dose: 0.75 g/L; initial concentration: 200 mg/L). Sharma et al. [244] prepared a gum tragacanth-cl-N,N-methylacrylamide/reduced graphene oxide (RGO) composite hydrogel by grafting N,N-dimethylacrylamide onto gum tragacanth in the presence of RGO and *N,N*″-methylene-*bis*-acrylamide through a free radical polymerization reaction (Figure 14). The microwave-assisted process may promote the reaction between gum tragacanth and the monomer in the presence of RGO to form a composite adsorbent. The results show that RGO is conducive to improving the adsorption capacity of the hydrogel, and so the composite adsorbent shows a high adsorption capacity of 666.6 mg/g and 473.9 mg/g for Hg(II) and Cr(VI) ions, respectively (initial concentration: 300 mg/L for Hg(II) and 500 mg/L for Cr(VI); adsorbent dose: 0.7 g/L for Hg(II) and 0.9 g/L for Cr(VI)). The adsorbents derived from gum tragacanth also show a superior removal effect for dye in water. Mallakpour et al. [245] prepared an eco-friendly nanocomposite adsorbent by compounding gum tragacanth with modified CaCO_3_ nanoparticles. The composite adsorbent shows a high adsorption capacity of 468.62 mg/g for methylene blue dye. Similarly, the composite hydrogel derived from gum tragacanth and CaCO_3_ nanoparticles can also effectively adsorb methylene blue in an aqueous solution, with a removal rate of 80% and a theoretical maximum adsorption capacity of 2145 mg/g [246]. Etemadinia et al. [247] synthesized a magnetic ZnFe_2_O_4_@SiO_2_@GT nanocomposite with an adsorption capacity of 109.37 mg/g for methylene blue. The adsorption of dye by the adsorbent is a spontaneous endothermic process, as confirmed by the thermodynamic parameters, such as enthalpy (ΔH = +9.36 kJ/mol), entropy (ΔS = +40.72 J/mol·k) and Gibbs free energy (ΔG = −3.5 kJ/mol). Sharma et al. [248] prepared a 2-hydroxyethyl methacrylate cross-linked gum tragacanth/TiO_2_ composite hydrogel through a microwave-assisted polymerization reaction route. The adsorbent can remove 99.3% of malachite green dye in water, and can be reused for more than five cycles.

In addition to water-soluble pollutants, the water pollution caused by water-insoluble oil pollutants should also be a concern. Saruchi et al. [249] prepared a gum tragacanth-grafted polymer adsorbent via a reaction among acrylic acid, methyl methacrylate, and gum tragacanth. The adsorbent has an oil adsorption capacity of 3.7 g/g, and can potentially be applied for the removal of oily pollutants from water.

### 3.10. Karaya Gum-Derived Adsorbents 

Karaya gum is an acidic polysaccharide with branch chains. The main chain in karaya gum is composed of α-(1→4)-linked D-galacturonic acid and α-(1→2)-linked L-aminoacyl residues. The side chain is connected by (1→3)-β-D-glucuronic acid, or (1→2) galacturonic acid unit β-D-galactose. Karaya gum can be used to synthesize new derivatives or materials, with an improved performance and expanded application through simple chemical modification, cross-linking, or graft polymerization routes. Karaya gum is capable of capturing heavy metal ions or dye molecules, so it is a good candidate for the preparation of eco-friendly adsorbents. It has been reported that karaya gum (Sterculia urens) has a good adsorption capability for mercury ions in aqueous solution (adsorption capacity: 62.5 mg/g), and 95% of the adsorbed metal ions can be recovered after desorption with 0.1 mol/L of HCl solution [250].

Karaya gum-based adsorbents have an excellent adsorption capacity towards dyes, which has been confirmed by many research works. The grafting polymerization modification of karaya gum may enhance its adsorption ability and yield a high-performance adsorption material. Mittal et al. [251] prepared a karaya gum-g-poly(acrylic acid-co-acrylamide)/SiO_2_ composite hydrogel adsorbent through the graft polymerization reaction route. The adsorbent can efficiently adsorb methylene blue from an aqueous solution, with an adsorption capacity of 1408.67 mg/g (initial concentration: 800 mg/L; adsorbent dose: 0.2 g/L) and a removal rate of 96% (adsorbent dose: 0.2 g/L; initial concentration: 200 mg/L) for methylene blue in water. Kumar et al. [252] synthesized a karaya gum-*g*-polyacrylamide/nickel sulfide nanoparticle composite adsorbent with a good adsorption capability towards rhodamine 6G dye in an aqueous solution (adsorption capacity: 1244.71 mg/g) (initial concentration: 500 mg/L; adsorbent dose: 0.4 g/L). The nanocomposite hydrogel adsorbents can be prepared by a graft polymerization reaction among karaya gum, silicon carbide and acrylic acid. The adsorbent shows excellent adsorption capacities of 757.57 and 497.51 mg/g for the cationic dyes malachite green (MG) and rhodamine B (RhB), respectively (adsorbent dose: 0.5 g/L for MG and 0.6 g/L for RhB; initial concentration: 1000 mg/L) [253]. Incorporating zeolite-Y into the karaya gum/poly(N-isopropylacylamide)/poly(acrylic acid) polymer matrix yields a composite adsorbent with an excellent dye adsorption capacity (1461.35 mg/g for Brilliant green) (initial concentration: 800 mg/L) [254]. In order to make the adsorbents easy to recycle, Ramakrishnan et al. [255] prepared a karaya gum/chitosan sponge with the adsorption capacities of 32.81 mg/g for methyl orange and 32.62 mg/g for methylene blue (initial concentration: 50 mg/L; adsorbent dose: 1.33 g/L). As the sponge-like adsorbent is blocky, it is easier to recover than powder adsorbent after use. Krishnappa et al. [256] synthesized a karaya gum-*graft*-poly(2-(dimethylamino) ethyl methacrylate) hydrogel through a radical polymerization reaction. The hydrogel adsorbent can spontaneously adsorb methylene blue and indigo carmine with the adsorption capacities of 89.28 and 101.42 mg/g, respectively. The polymerization reaction rate and grafting efficiency can be improved by a microwave-assisted process. Preetha et al. [198] synthesized a karaya gum-g-poly((2-methyloyloxy ethyl) trimethyl ammonium chloride)/montmorillonite composite adsorbent capable of adsorbing dye through a microwave-assisted radical polymerization reaction. The results showed that the nanocomposite adsorbents can spontaneously adsorb methylene blue, toluidine blue, crystal violet and azure B dyes, with adsorption capacities of 155.85 mg/g, 149.64 mg/g, 137.77 mg/g and 128.78 mg/g, respectively. Pandey et al. [257] prepared a karaya gum crosslinked poly(acrylamide-co-acrylonitrile)@silver nanoparticles nanocomposite hydrogel by by an in situ crosslinking copolymerization reaction between acrylamide and acrylic acid in a dispersion of AgNPs, stabilized with karaya gum. In the reaction process, the initiator may ignite karaya gum and monomers to generate free radicals, and then the grafting and chain propagation reaction is carried out to form a karaya gum-grafted polymer. The resultant hydrogel can adsorb efficiently crystal violet dye by a strong hydrogen bonding interaction, dipole–dipole interaction and electrostatic interaction with a high removal rate of 99% and adsorption capacity of 1000 mg/g (initial concentration: 500 mg/L; adsorbent dose: 0.2 g/L), and the adsorbent can be reused at least ten times after use. The semi-interpenetrating network hydrogel adsorbent consisting of a fenugreek karaya gum-g-polyacrylamide and polyvinyl alcohol can be prepared through a microwave initiation polymerization process [258]. The adsorbent shows the adsorption capacities of 82.28, 72.94, 48.75, and 34.67 mg/g for the cationic dyes methylene blue, crystal violet, rhodamine B, and toluidine blue, respectively.

### 3.11. Gum Ghatti-Derived Adsorbents 

Gum ghatti is the exudate of a broad-leaved Anogeissus latifolia tree, belonging to the Combretaceae family. Gum ghatti is a biodegradable anionic polysaccharide with a complex structure. The carboxylic and hydroxyl groups of gum ghatti mean it has excellent gelling, thickening, emulsifying and surface activities, and can capture metal ions by electrostatic attraction and a complexing interaction. These advantages of gum ghatti make it a potential gum to be used as a raw material for the preparation of new adsorption materials with excellent performance. Singha et al. [259] prepared a gum ghatti-g-(N-isopropylacrylamide-co-3-(N-isopropylacrylamido) protonic acid-co-sodium acrylate) hydrogel adsorbent and evaluated its adsorption performance for heavy metals. The results show that the hydrogel adsorbent has high adsorption capacities, of 1477.83, 1568.81, 1582.38 and 1518.09 mg/g for Cd(II), Pb(II), Bi(III), and Sb(III), respectively. In order to make the adsorbent easy to separate from the solution by an external magnetic field after use, Kulal and Badalamoore [260] prepared a magnetic Gum ghatti-g-4-acryloylmorphine/Fe_3_O_4_ composite adsorbent with an adsorption capacity of 249.9 mg/g for Cu(II) and 235.1 mg/g for Hg(II). The adsorbent also shows excellent regeneration ability after being desorbed with an acidic solution (pH 1.2), and the desorption rate is higher than 97%. This proves that the composite adsorbent is suitable for the removal and recovery of heavy metals from an aqueous solution.

With the increasing attention paid to the harmless treatment of printing and dyeing wastewater, gum ghatti-based adsorbents have received much attention for dye removal. A series of adsorption materials that are suitable for removing different types of dyes can be obtained through the graft copolymerization reaction of gum ghatti with monomers. Mittal et al. [261] prepared a magnetic ghatti gum-*g*-poly(acrylic acid-co-acrylamide)/Fe_3_O_4_ composite adsorbent capable of adsorbing Rhodamine B. The maximum adsorption capacity of the adsorbent for the Rhodamine B dye reaches 654.87 mg/g, and a better adsorption capacity was attained after being reused for three cycles. Mittal et al. [262] prepared a magnetic Gum ghatti-cl-poly(acrylic acid)/Fe_3_O_4_ nanocomposite adsorbent for the removal of dye pollutants in water. The adsorbent can be recycled continuously, and has a high adsorption capacity of 671.14 mg/g for methylene blue dye. Lohar and Joshi [263] synthesized a ghatti gum-*graft*-poly(acrylamide) polymer adsorbent by the gamma-ray induced radical polymerization reaction route. The adsorbent can efficiently adsorb uranium ions and thorium ions (adsorption capacity: 367.65 mg/g and 125.95 mg/g, respectively) by an ion exchange process. Kankeu et al. [264] reported that the gum ghatti-*graft*-poly(acrylic acid) adsorbent is able to adsorb methylene blue and rhodamine B with the adsorption capacities of 909.09 mg/g and 819.67 mg/g, respectively. The maximum removal rate also reached 99% and 98%, respectively. Goddeti et al. [265] synthesized a gum ghatti-*graft*-poly(acrylamide)/zero valent iron composite adsorbent with a high adsorption capacity of 250 mg/g for Congo red. Makhado et al. [266] prepared a composite adsorption material by incorporating titanium dioxide into gum ghatti-*graft*-poly(acrylic acid) for the adsorption of malachite green. The titanium dioxide plays a positive role in reducing the swelling rate of the adsorbent, and improving the mechanical strength and adsorption performance of the adsorbent. The saturation adsorption capacity of the adsorbent reaches 2145 mg/g, and the adsorption capacity remains good after being recycled more than 5 cycles.

### 3.12. Galactomannan Gum and Fenugreek Gum-Derived Adsorbents 

**Figure 15:**
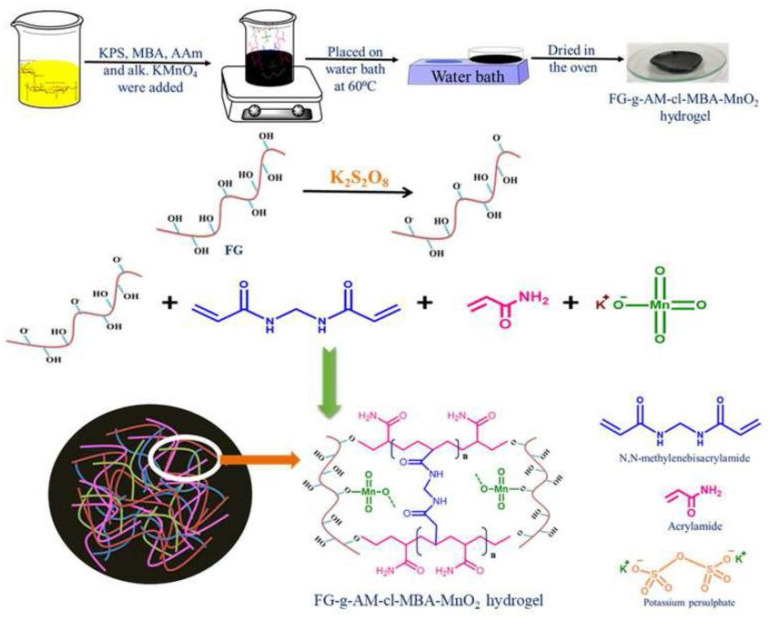
Schematic illustration of the synthesis mechanism of FG-g-AM-cl-MBA-MnO_2_ hydrogel [267].

Galactomannan gum was used as a candidate to prepare adsorption materials for the removal of pollutants. Graft polymerization is often used to prepare galactomannan gum-based adsorption materials. Sharma et al. [244] prepared a gum tragacanth-cl-N,N-dimethyllacrylamide/reduced graphene oxide (GT-cl-poly (DMA)/RGO) composite adsorbent. Under the optimized conditions, the adsorbent shows a high adsorption capacity of 666.6 mg/g and 473.9 mg/g for Hg(II) and Cr(VI) ions, respectively. In addition to being able to adsorb heavy metals, galactomannan gum-based materials also show good adsorption properties for dyes. Sharma et al. [267] prepared a fenugreek gum-*graft*-poly(acrylamide-cl-methylenebisacrylamide (MBA)/MnO_2_ (FG-g-AM-cl-MBA-MnO_2_) hydrogel through a radical polymerization-crosslinking reaction (Figure 15). In this process, 0.5 g of fenugreek gum (FG) was dissolved in 18.25 mL distilled water to form a uniform dispersion; then, 0.025 g potassium persulfate, 2.5 g acrylamide and 0.05 g MBA, 6.75 mL 0.25 M alkaline KMnO_4_ solution were added into the solution while stirring for 60 min. Finally, the reaction mixture was placed in a 60 °C water bath until the gel was formed. The hydrogel shows a high removal rate of 98.5% (for malachite green) and 96.3% (for methylene blue), and adsorption capacities (526.31 mg/g for malachite green and 483.09 mg/g for methylene blue).

## 4. Conclusions, Perspectives, and Future Perspectives

Green development, environmental safety, and health have become the focus of attention across the world. However, the increasingly serious nature of water pollution has become a difficult problem faced by human beings. It is urgent to develop effective materials or technologies to eliminate the harm caused by water pollution. In recent years, adsorption materials, that can effectively remove pollutants, have been successfully developed and used for the adsorption and removal of various pollutants. With more and more attention paid to the safety of materials, adsorption materials prepared from natural starting materials have become a new favorite in this field; these can achieve the purification of wastewater under the premise of minimizing secondary pollution. The renewable and non-toxic advantages of natural plant gums make them a potential for preparing green high-performance adsorption materials; plant gums will attract more attention in the adsorption and removal of various contaminants. In this paper, the preparation of natural plant gums-based adsorbents and their applications in pollutant removal were summarized systematically. Through the comparative analysis of different adsorbents derived from plant gums (i.e., guar gum, xanthan gum, pectin, carrageenan, gum tragacanth, karaya gum), it can be concluded that the adsorption performance of all natural plant gums-based adsorbents can be improved by modification, compounding with other components, or graft copolymerization routes; the graft polymerization of plant gums is more effective in improving the adsorption performance, and the plant gum containing the carboxyl group is more suitable for preparing gums-based adsorption materials. The introduction of functional groups into plant gums can improve their adsorption capacities for pollutants, especially for heavy metal ions. However, the current research on natural plant gums-based adsorption materials is limited to the removal of a few conventional pollutants, such as heavy metals, dyes, etc., and the attention paid to emerging pollutants is still lacking. The modification methods of plant gums are limited, so more green methods, such as radiation synthesis, should be developed to modify natural plant gums; in addition, adsorption materials with new structures should be designed and constructed. It is necessary to develop adsorption materials with all natural components to minimize the use of synthetic chemicals, and to develop new methods for the regeneration and reutilization of plant gums-based adsorption materials to maximize their usage values. It is also necessary to design and construct multi-functional natural plant gums-based adsorption materials to meet the purifying needs of increasingly complex wastewaters. It is expected that this paper can provide a useful reference for the design, preparation and application of more natural plant gums-based adsorption materials.

## Data Availability

The data presented in this study are available on request from the corresponding author.
